# SpaHDSRL: hierarchical dual-graph self-supervised representation learning for integrating spatially resolved multi-omics data

**DOI:** 10.1093/bib/bbag517

**Published:** 2026-09-18

**Authors:** Xiang Li, Kangkang Zhang, Yifei Li, Fangrong Yan, Bosheng Li, Qian Ding

**Affiliations:** Research Center of Biostatistics and Computational Pharmacy, China Pharmaceutical University, Nanjing 211198, Jiangsu Province, China; Research Center of Biostatistics and Computational Pharmacy, China Pharmaceutical University, Nanjing 211198, Jiangsu Province, China; Research Center of Biostatistics and Computational Pharmacy, China Pharmaceutical University, Nanjing 211198, Jiangsu Province, China; Research Center of Biostatistics and Computational Pharmacy, China Pharmaceutical University, Nanjing 211198, Jiangsu Province, China; Research Center of Biostatistics and Computational Pharmacy, China Pharmaceutical University, Nanjing 211198, Jiangsu Province, China; Research Center of Biostatistics and Computational Pharmacy, China Pharmaceutical University, Nanjing 211198, Jiangsu Province, China

**Keywords:** spatial omics, multi-omics integration, dual-graph Modeling, hierarchical fusion, spatial domain identification

## Abstract

Spatial multi-omics technologies facilitate simultaneous measurement of multiple molecular modalities within their native spatial context, offering opportunities to characterize tissue organization and cellular heterogeneity. However, effective integration remains challenging because such data concurrently encode spatial adjacency and molecular similarity, while also being limited by high dimensionality, sparsity, noise, and cross-modality heterogeneity. Here, we propose SpaHDSRL, a hierarchical dual-graph self-supervised representation learning framework for spatial multi-omics integration. SpaHDSRL jointly models a shared spatial graph and modality-specific feature graphs, which are integrated through an adaptive gated hierarchical fusion strategy to learn coherent and informative latent representation. To further enhance representation quality, SpaHDSRL combines a Deep Graph Infomax-based objective with spatial regularization, preserving both global informativeness and local spatial consistency. Experiments on simulated and real datasets demonstrate that SpaHDSRL consistently achieves superior performance over existing methods in both the accuracy and robustness of spatial domain identification. Downstream analyses further highlight its utility in marker discovery, functional enrichment, second-modality-associated analysis, and cell–cell communication inference, underscoring its value for dissecting tissue architecture, developmental programs, and multicellular interactions in complex biological systems. The source code of SpaHDSRL is available at https://github.com/Lisa62103/SpaHDSRL.

## Introduction

Recent advances in spatially resolved omics technologies have enabled the simultaneous measurement of molecular profiles and their spatial context, providing unprecedented opportunities to investigate tissue organization and elucidate complex cellular interactions *in situ* [1, 2]. Traditional single-modality measurements capture limited molecular information from only one aspect. In contrast, spatial multi-omics data integrate complementary information across multiple molecular layers, thereby providing a more comprehensive view of biological systems and facilitating the elucidation of tumor progression and microenvironmental organization [3]. Recent computational efforts, such as scBSP, have also highlighted the importance of identifying spatially organized molecular signals from high-resolution spatial omics data, which provide useful information for characterizing tissue architecture and local regulatory programs [4]. Consequently, the effective integration of such heterogeneous data to extract deeper biological insights has become a critical step toward understanding the molecular and spatial principles that govern complex tissues.

Despite the promising potential of spatial multi-omics integration, its inherent complexity poses substantial challenges. A core difficulty is that such data involve two complementary forms of structure: spatial adjacency, which reflects local tissue organization, and molecular similarity, which captures shared cellular states or functional programs [5]. These two structures provide distinct yet mutually informative views of tissue organization. Relying solely on spatial proximity may obscure critical molecular heterogeneity among nearby regions (e.g. transcriptomic differences among distinct cell types within the same spatial locale), whereas relying only on molecular similarity may disrupt spatial continuity and tissue architecture (e.g. functionally similar cells might be physically distant without direct spatial connection). Furthermore, different omics modalities often contain both shared and modality-specific information [6, 7], making it challenging to derive unified representations without losing biologically meaningful signals. The high dimensionality, sparsity, and noise inherent in spatial multi-omics data further increase the complexity of integration [8, 9]. To address these challenges, various computational methods have been developed for multi-omics integration and spatial domain identification. However, existing approaches generally exhibit several limitations in handling spatial multi-omics data. Some methods primarily focus on aligning cross-modality data into a shared low-dimensional space, for instance, through Procrustes analysis or deep learning techniques such as Pamona [10], WNN [11], and MultiVI [12]. While effective for feature integration, these methods may not fully exploit spatial structures, which can limit their ability to accurately distinguish complex tissue regions. Other methods, such as COSMOS [13], incorporate spatial information by constructing a single graph representation to capture adjacency relationships between cells. Recent image-assisted methods, such as stDyer-image [14], further demonstrate that additional tissue-context information can improve spatial domain identification by incorporating morphological features into clustering analysis. Nevertheless, these methods do not fully address the heterogeneity across different modalities or the effective fusion of shared and modality-specific information in paired spatial multi-omics data. Even method such as SpatialGlue [5] that attempt to consider both spatial and molecular information adopt relatively shallow fusion strategies, which may struggle to capture cross-modal complementarity while preserving local neighborhood structures within each modality. Furthermore, current approaches generally lack robust self-supervised objectives and explicit spatial constraints for learning biologically meaningful unified representations.

To overcome the aforementioned limitations, we propose SpaHDSRL, a hierarchical dual-graph self-supervised representation learning framework for spatial multi-omics integration. SpaHDSRL jointly models a shared spatial graph and modality-specific feature graphs to capture tissue organization and molecular similarity from complementary perspectives. Based on this dual-graph design, SpaHDSRL uses an adaptive hierarchical gated fusion strategy to integrate spatial and molecular representations within each modality and then aggregate complementary information across modalities into a unified latent representation. In addition, a Deep Graph Infomax-based self-supervised objective and a spatial regularization term are incorporated to enhance representation learning and preserve spatial coherence. For evaluation, SpaHDSRL is compared with existing algorithms on both simulated and real spatial multi-omics datasets, consistently achieving improved performance in spatial domain identification, latent representation learning, and robustness under noisy and perturbed settings. Moreover, downstream analyses, including marker gene characterization, functional enrichment, second-modality-associated analysis, and cell–cell communication inference, further demonstrates that the SpaHDSRL-based representation supports biologically meaningful interpretation across diverse tissue contexts.

## Methods

### Data preprocessing

We evaluated SpaHDSRL on two simulated datasets and three real spatial multi-omics datasets. For the first simulated dataset, paired spatial transcriptomic and ADT profiles were generated following the simulation strategies described in previous studies [5, 9], followed by modality-specific normalization. For the second simulated dataset, we used a 10× Genomics Visium human breast cancer spatial transcriptomics dataset [15] to simulate an RNA-protein setting based on fine-grained tissue annotations. Specifically, spot-level expression profiles were randomly shuffled within the invasive ductal carcinoma 5 (IDC 5)/IDC 4 regions for the first modality and within the IDC 5/IDC 2 regions for the second modality. Gaussian multiplicative noise was then added to the shuffled matrices to generate paired modalities with complementary spatial information. Both simulated modalities were subsequently normalized.

For real-data evaluation, we collected three mouse spatial multi-omics datasets from embryonic brain [16], whole embryo [17], and thymus tissue [18], corresponding to RNA-protein, RNA-ATAC, and RNA-ADT modalities, respectively. The details of these datasets are summarized in Table S1. For the RNA modality, low-quality pixels with fewer than 200 detected genes and genes expressed in fewer than 10 pixels were removed. Highly variable genes were then selected using the Seurat v3 method [19], followed by library-size normalization and log transformation using SCANPY [20]. For the ATAC modality, chromatin accessibility signals were aggregated into 100-kilobase (kb) genomic bins to generate a sparse accessibility matrix, followed by term frequency-inverse document frequency (TF-IDF) transformation and latent semantic indexing (LSI)-based dimensionality reduction. After removing the first LSI component, the remaining 50 components were retained for downstream analysis. Only pixels shared by both modalities were kept. For the DBiT-seq mouse embryonic brain dataset and Stereo-CITE-seq mouse thymus dataset, we followed the preprocessing protocols used in previous studies [5, 13].

### Spatial and modality-specific graph construction

SpaHDSRL is designed for paired spatial omics modalities, such as RNA-protein or RNA-chromatin accessibility data, that are measured at an identical set of spatial locations. Let the two input matrices be denoted as ${\mathbf{X}}^{(1)}\in{\mathbb{R}}^{n\times{p}_1}$ and ${\mathbf{X}}^{(2)}\in{\mathbb{R}}^{n\times{p}_2}$ where *n* is the number of spatial locations, ${p}_1$ and ${p}_2$ are the feature dimensions of the two modalities, respectively. Importantly, both modalities share the same spatial coordinates, each spatial location jointly yields measurements in both feature spaces.

To jointly capture spatial dependency and within-modality molecular similarity, we construct three *k*-nearest-neighbor (KNN) graphs: a spatial graph ${G}_s=\left(\mathcal{V},{\mathcal{E}}_s\right)$ based on coordinates, and two modality-specific feature graphs ${G}_f^{(1)}=\left(\mathcal{V},{\mathcal{E}}_f^{(1)}\right)$ and ${G}_f^{(2)}=\left(\mathcal{V},{\mathcal{E}}_f^{(2)}\right)$ based on the molecular profiles of the two modalities. The corresponding graph structures are denoted by ${\mathbf{A}}_s$, ${\mathbf{A}}_f^{(1)}$ and ${\mathbf{A}}_f^{(2)}$, respectively. Each adjacency matrix is constructed by identifying the *k* nearest neighbors of each node using Euclidean distance in the corresponding space, where an entry is set to 1 if two nodes are connected under this criterion, and 0 otherwise. Specifically, let ${c}_i$ denote the spatial coordinate of the *i*-th spatial location, and let ${\mathcal{N}}_k^s(i)$ be the set of its k nearest neighbors in the coordinate space. The spatial adjacency matrix ${\mathbf{A}}_s\in{\mathbb{R}}^{n\times n}$ is defined as


(1) \begin{eqnarray*} {\left({\mathbf{A}}_s\right)}_{ij}=\left\{\begin{array}{@{}ll}1,& j\in{\mathcal{N}}_k^s(i),\\{}0,& \mathrm{otherwise}.\end{array}\right. \end{eqnarray*}

For the *m*-th modality ($m\in \left\{1,2\right\}$), let ${\mathbf{x}}_i^{(m)}$ denote the feature vector of the *i*-th spatial location in ${\mathbf{X}}^{(m)}$, and let ${\mathcal{N}}_k^{f,m}(i)$ be the set of its k-nearest neighbors in the corresponding molecular feature space. The modality-specific feature adjacency matrix ${\mathbf{A}}_f^{(m)}\in{\mathbb{R}}^{n\times n}$ is defined as


(2) \begin{eqnarray*} {\left({\mathbf{A}}_f^{(m)}\right)}_{ij}=\left\{\begin{array}{@{}ll}1,& j\in{\mathcal{N}}_k^{f,m}(i),\\{}0,& \mathrm{otherwise}.\end{array}\right. \end{eqnarray*}

In our implementation, the same KNN parameter k was used for both the spatial graph and modality-specific feature graphs, and the specific k values used in different experiments are reported in Supplementary S1. The KNN graphs were directly converted into edge-index format for subsequent GCN propagation without additional manual symmetrization.

### Dual graph encoder within the modality

The spatial graph and the feature graph characterize two complementary types of neighborhood relationships. Specifically, the spatial graph captures local proximity patterns in the physical space, whereas the feature graph reflects similarity relationships in the molecular feature space. To fully exploit these two distinct structural views, we employ two parallel graph convolution paths for each modality. One path is constructed on the spatial graph to capture spatially contextualized representations, while the other is built on the feature graph to learn representations that preserve molecular similarity among spatial spots. For the *m*-th modality ($m\in \left\{1,2\right\}$), the spatial graph convolution path is defined as


(3) \begin{eqnarray*} {\mathbf{H}}_{s,1}^{(m)}=\varphi \left({\mathrm{GCN}}_{s,1}^{(m)}\left({\mathbf{X}}^{(m)},{\mathbf{A}}_s\right)\right), \end{eqnarray*}

(4) \begin{eqnarray*} {\mathbf{H}}_{s,2}^{(m)}=\varphi \left({\mathrm{GCN}}_{s,2}^{(m)}\left({\mathbf{H}}_{s,1}^{(m)},{\mathbf{A}}_s\right)\right). \end{eqnarray*}

Where ${\mathbf{X}}^{(m)}$ denotes the input feature matrix of the *m*-th modality, ${\mathbf{A}}_s$ denotes the shared spatial adjacency matrix, and $\varphi \left(\cdotp \right)$ denotes the PReLU activation function. By aggregating information from spatially adjacent neighbors, the two-layer graph convolutional network (GCN) encoder enables the model to capture local spatial dependencies and contextual interactions. To further stabilize the learned representation and ensure comparability across spatial spots, the resulting embeddings are subjected to normalization:


(5) \begin{eqnarray*} {\mathbf{Z}}_s^{(m)}=\operatorname{norm}\left({\mathbf{H}}_{s,2}^{(m)}\right). \end{eqnarray*}

Where $\operatorname{norm}\left(\cdotp \right)$ denotes row-wise ${L}_2$ normalization. Meanwhile, we construct a feature-graph path with the same overall design, but operating on the feature graph so as to propagate information among molecularly similar nodes. The feature path is defined as:


(6) \begin{eqnarray*} {\mathbf{H}}_{f,1}^{(m)}=\varphi \left({\mathrm{GCN}}_{f,1}^{(m)}\left({\mathbf{X}}^{(m)},{\mathbf{A}}_f^{(m)}\right)\right), \end{eqnarray*}

(7) \begin{eqnarray*} {\mathbf{H}}_{f,2}^{(m)}=\varphi \left({\mathrm{GCN}}_{f,2}^{(m)}\left({\mathbf{H}}_{f,1}^{(m)},{\mathbf{A}}_f^{(m)}\right)\right), \end{eqnarray*}

(8) \begin{eqnarray*} {\mathbf{Z}}_f^{(m)}=\operatorname{norm}\left({\mathbf{H}}_{f,2}^{(m)}\right). \end{eqnarray*}

Where ${\mathbf{A}}_f^{(m)}$ denotes the modality-specific feature adjacency matrix of the current modality.

Although the spatial and feature paths within the same modality take the same input matrix ${\mathbf{X}}^{(m)}$, they use different adjacency matrices and independent GCN parameters. This allows the two convolution paths to learn graph-specific representations associated with physical spatial proximity and molecular feature similarity respectively. Through the dual-path design, each modality simultaneously captures spatial-context and feature-similarity information from two complementary perspectives, thereby yielding two base embeddings consisting of a spatial embedding ${\mathbf{Z}}_{\mathrm{s}}^{(m)}$ and a feature embedding ${\mathbf{Z}}_{\mathrm{f}}^{(m)}$.

### Intra-modality gated neighbor aggregation fusion

Using only the spatial graph may lead to over-smoothed representations and thus weaken fine-grained local biological signals, whereas relying solely on the feature graph may capture molecular similarity at the expense of structural constraints imposed by tissue organization. Therefore, we introduce a gated neighbor aggregation fusion module to adaptively integrate the two graph-derived embeddings within each modality. The key idea is to use one view to retrieve informative neighbors from the other view, and then determine the relative contribution of the two views via spot-specific gating.

Given the spatial embedding ${\mathbf{Z}}_s$ and the feature embedding ${\mathbf{Z}}_f$, we first measure their cross-view consistency by constructing a cross-view similarity matrix:


(9) \begin{eqnarray*} \boldsymbol{\Omega} =\operatorname{norm}\left({\mathbf{Z}}_s\right)\operatorname{norm}{\left({\mathbf{Z}}_f\right)}^{\mathrm{T}}\in{\mathbb{R}}^{n\times n}. \end{eqnarray*}

Here, each entry of $\boldsymbol{\Omega}$ reflects the similarity between a spot represented in the spatial branch and another spot represented in the feature branch. In this way, the similarity matrix serves as a bridge between the two views, allowing the model to identify potentially useful cross-view neighbors rather than restricting aggregation to a single graph structure.

For each spot *i*, we select the top-*k* most similar cross-view neighbors according to the *i*-th row of $\boldsymbol{\Omega}$:


(10) \begin{eqnarray*} {\mathcal{N}}_i=\mathrm{TopK}\left({\boldsymbol{\Omega}}_{i,:},k\right). \end{eqnarray*}

The neighborhood size *k* controls the number of cross-view neighbors included in the aggregation. Since a larger *k* may include more weakly related spots, we kept *k* within 0.5% of the total number of spots in each dataset and further assessed its effect on neighbor similarity and clustering performance in the sensitivity analysis (Table S13). To further distinguish the relative importance of the selected neighbors, we convert their similarity scores into normalized weights through a temperature-scaled softmax:


(11) \begin{eqnarray*} {w}_{ij}=\frac{\exp \left({\Omega}_{ij}/\gamma \right)}{\sum_{l\in{\mathcal{N}}_i}\exp \left({\Omega}_{il}/\gamma \right)},\kern0.5em j\in{\mathcal{N}}_i. \end{eqnarray*}

Where the temperature parameter *γ* controls the sharpness of the weight distribution. Because the weights are normalized from cross-view similarity scores, neighbors with lower similarity receive smaller contributions during aggregation, reducing the influence of weakly related spots. These weights are then used to aggregate neighbor representations from the feature branch:


(12) \begin{eqnarray*} {\hat{\mathbf{z}}}_i=\sum_{j\in{\mathcal{N}}_i}{w}_{ij}{\mathbf{z}}_{f,j}. \end{eqnarray*}

Meanwhile, instead of using a fixed fusion coefficient for all spots, we generate an adaptive gating coefficient from the spatial embedding:


(13) \begin{eqnarray*} {\boldsymbol{\alpha}}_i=\sigma \left({\mathbf{W}}_g{\mathbf{z}}_{s,i}+{\mathbf{b}}_g\right). \end{eqnarray*}

Where $\sigma \left(\cdotp \right)$ denotes the sigmoid activation function, and the gating coefficient ${\boldsymbol{\alpha}}_i$ determines the relative contribution of the original spatial representation and the aggregated cross-view representation for spot *i*. Since ${\boldsymbol{\alpha}}_i$ is learned independently for each spot, the model can dynamically adjust the fusion strategy according to local structural context and representation quality.

The fused representation of this modality is then defined as ${\mathbf{z}}_i={\boldsymbol{\alpha}}_i{\mathbf{z}}_{s,i}+\left(\mathbf{1}-{\boldsymbol{\alpha}}_i\right){\hat{\mathbf{z}}}_i$. Collecting all spots gives the within-modality fused embedding $\mathbf{Z}={\left[{\mathbf{z}}_1,\dots, {\mathbf{z}}_n\right]}^{\mathrm{T}}\in{\mathbb{R}}^{n\times d}$, which allows the model to adaptively balance spatial-context information and molecular-similarity information on a per-spot basis, rather than relying on a fixed fusion coefficient.

### Cross-modality hierarchical fusion

After consolidating the information within each modality, we further integrate the two modalities in the latent space to obtain a unified cross-modal representation. Let ${\mathbf{Z}}^{(1)}={\left[{\mathbf{z}}_1^{(1)},\dots, {\mathbf{z}}_n^{(1)}\right]}^{\mathrm{T}}$ and ${\mathbf{Z}}^{(2)}={\left[{\mathbf{z}}_1^{(2)},\dots, {\mathbf{z}}_n^{(2)}\right]}^{\mathrm{T}}$ denote the within-modality fused embeddings of the first and second omics layers, respectively. We then apply the same gated neighbor aggregation strategy across modalities by treating one modality as the reference view and the other as the complementary view. Specifically, we compute the cross-modal similarity matrix:


(14) \begin{eqnarray*} {\boldsymbol{\Omega}}^{\left(1,2\right)}=\operatorname{norm}\left({\mathbf{Z}}^{(1)}\right)\operatorname{norm}{\left({\mathbf{Z}}^{(2)}\right)}^{\mathrm{T}}. \end{eqnarray*}

For each spot *i*, we select its top-*k* neighbors in the second modality according to the *i*-th row of ${\boldsymbol{\Omega}}^{\left(1,2\right)}$:


(15) \begin{eqnarray*} {\mathcal{N}}_i^{\left(1,2\right)}=\mathrm{TopK}\left({\boldsymbol{\Omega}}_{i,:}^{\left(1,2\right)},k\right). \end{eqnarray*}

Similar to the intra-modality fusion described above, the selected cross-modal neighbors are assigned similarity-based weights and then aggregated to form a complementary representation from the second modality. A spot-specific cross-modal gating coefficient is then introduced to adaptively integrate this complementary representation with the original first-modality embedding.


(16) \begin{eqnarray*} {w}_{ij}^{\left(1,2\right)}=\frac{\exp \left({\Omega}_{ij}^{\left(1,2\right)}/\gamma \right)}{\sum_{l\in{\mathcal{N}}_i^{\left(1,2\right)}}\exp \left({\Omega}_{il}^{\left(1,2\right)}/\gamma \right)}. \end{eqnarray*}

(17) \begin{eqnarray*} {\hat{\mathbf{z}}}_i^{\left(1,2\right)}=\sum_{j\in{\mathcal{N}}_i^{\left(1,2\right)}}{w}_{ij}^{\left(1,2\right)}{\mathbf{z}}_j^{(2)}. \end{eqnarray*}

(18) \begin{eqnarray*} {\boldsymbol{\alpha}}_i^{\left(1,2\right)}=\sigma \left({\mathbf{W}}_c{\mathbf{z}}_i^{(1)}+{\mathbf{b}}_c\right). \end{eqnarray*}

The final joint representation of spot *i* is calculated as ${\mathbf{z}}_i^{\ast }={\boldsymbol{\alpha}}_i^{\left(1,2\right)}{\mathbf{z}}_i^{(1)}+\left(\mathbf{1}-{\boldsymbol{\alpha}}_i^{\left(1,2\right)}\right){\hat{\mathbf{z}}}_i^{\left(1,2\right)}$, and collecting all spots yields the unified embedding ${\mathbf{Z}}^{\ast }={\left[{\mathbf{z}}_1^{\ast },\dots, {\mathbf{z}}_n^{\ast}\right]}^{\mathrm{T}}\in{\mathbb{R}}^{n\times d}$. Based on that, the two modalities are integrated across omics layers to produce a shared latent representation.

In the experiments, RNA was used as the first modality when available, and the corresponding ATAC, ADT, or protein modality was used as the second modality. This setting was motivated by the fact that RNA profiles generally provide a broad and continuous transcriptomic representation of tissue organization, covering diverse gene-expression programs related to cell identity and spatial transcriptional patterning. In contrast, ATAC, ADT, and protein modalities provide more targeted but complementary information, such as regulatory accessibility or protein-level cell-state signals. Therefore, using RNA as the reference modality allows SpaHDSRL to establish a relatively stable global tissue structure, while the second modality contributes complementary regulatory or protein-associated information during cross-modality fusion. To evaluate the influence of modality order, we further performed modality-swapping experiments, with the results reported in Tables S17 and S18.

### Self-supervised graph information maximization

Since reliable cell labels are often unavailable in spatial multi-omics datasets, we train the encoder in a self-supervised manner. Specifically, we adopt a Deep Graph Infomax (DGI) objective to maximize the agreement between local node representations and global graph semantics [21]. Let the positive and negative embeddings be denoted by ${\mathbf{Z}}^{+}=\mathrm{Encoder}\left({\mathbf{X}}^{(1)},{\mathbf{X}}^{(2)},{\mathbf{A}}_s,{\mathbf{A}}_f^{(1)},{\mathbf{A}}_f^{(2)}\right)$ and ${\mathbf{Z}}^{-}=\mathrm{Encoder}\left({\overset{\sim }{\mathbf{X}}}^{(1)},{\overset{\sim }{\mathbf{X}}}^{(2)},{\mathbf{A}}_s,{\mathbf{A}}_f^{(1)},{\mathbf{A}}_f^{(2)}\right)$, respectively. Here, to generate ${\overset{\sim }{\mathbf{X}}}^{(1)}$ and ${\overset{\sim }{\mathbf{X}}}^{(2)}$, we perturb the input features of both modalities using the same random permutation, which disrupts the correspondence between node features and the original graph structure while preserving the one-to-one pairing between the two omics modalities. This corruption operation helps to perturb node features while keeping graph structures unchanged, encouraging the encoder to distinguish consistent node-graph associations from corrupted ones.

A bilinear discriminator is then used to evaluate the agreement between a node embedding and the global summary:


(19) \begin{eqnarray*} D\left({\mathbf{z}}_i,\mathbf{s}\right)=\sigma \left({\mathbf{z}}_i^{\mathrm{T}}\mathbf{Ws}\right). \end{eqnarray*}

Where $\mathbf{W}\in{\mathbb{R}}^{d\times d}$ is a learnable parameter matrix, and $\mathbf{s}\in{\mathbb{R}}^d$ is a global summary of the positive embedding output, obtained through a mean readout over all nodes. The self-supervised objective is then defined as


(20) \begin{eqnarray*} \kern1.75em {\mathcal{L}}_{\mathrm{DGI}}=-\frac{1}{n}\sum_{i=1}^n\left[\log D\left({\mathbf{z}}_i^{+},\mathbf{s}\right)+\log \left(1-D\left({\mathbf{z}}_i^{-},\mathbf{s}\right)\right)\right]. \end{eqnarray*}

This objective encourages the learned representation to preserve coherent graph-level semantics while suppressing spurious agreement induced by corrupted inputs.

### Spatial regularization

In addition to incorporating the spatial graph into message passing, we further impose a spatial regularization term to preserve global spatial organization in the latent space. Let ${d}_z\left(i,j\right)$ denote the Euclidean distance between samples *i* and *j* in the joint embedding ${\mathbf{Z}}^{\ast }$, and let ${d}_s\left(i,j\right)$ denote the Euclidean distance between their spatial coordinates. Both latent and spatial distances were normalized to [0,1], and the spatial regularization term was defined as


(21) \begin{eqnarray*} {\mathcal{L}}_{\mathrm{sp}}=\frac{1}{\left|\mathcal{P}\right|}\sum_{\left(i,j\right)\in \mathcal{P}}\left(1-{\tilde{d}}_z\left(i,j\right)\right){\tilde{d}}_s\left(i,j\right) \end{eqnarray*}

Where ${\tilde{d}}_z\left(i,j\right)$ and ${\tilde{d}}_S\left(i,j\right)$ are the normalized latent and spatial distances respectively, and $\mathcal{P}$ denotes the spot set used for computing the spatial regularization term. In our experiments, $\mathcal{P}$ was constructed from the full set of spots in each dataset. The final objective of the model is


(22) \begin{eqnarray*} \mathcal{L}={\mathcal{L}}_{\mathrm{DGI}}+\lambda{\mathcal{L}}_{\mathrm{sp}} \end{eqnarray*}

The parameter *λ* controls the strength of the spatial regularization. In practice, its value was selected to balance spatial coherence and representation flexibility. For datasets with relatively intact and spatially continuous tissue domains, a larger *λ* was adopted to encourage better preservation of the physical spatial organization in the learned latent representation. For datasets with more complex spatial structures or higher noise levels, a moderate *λ* was used to avoid imposing overly strict spatial constraints. The specific *λ* values employed in our experiments, along with the corresponding sensitivity analysis, are provided in Supplementary S1 and Table S15.

### Domain segmentation and downstream analysis

Domain segmentation was performed on the low-dimensional embeddings using a unified KNN-Leiden clustering workflow. For each dataset, the embedding learned by each method was used as input to construct a KNN graph with the same number of 50 neighbors, followed by Leiden clustering over the same resolution search range. The target number of clusters, clustering procedure, and evaluation metrics were kept consistent across methods within the same dataset. For simulated datasets, the Leiden resolution was selected according to the highest silhouette coefficient of the learned embedding, and ground-truth labels were used only for final quantitative evaluation. For real datasets, the resolution was selected according to the expected number of spatial domains reported in the original studies or prior biological annotations. Sensitivity analysis of downstream KNN graph size is provided in Table S14. The resulting Leiden clusters were used as the final domain assignments for downstream analyses, including visualization, marker gene identification, Gene Ontology (GO) enrichment [22], second-modality-associated analysis, and cell–cell communication analysis [23].

### Ablation and sensitivity analyses

To evaluate the contribution of key SpaHDSRL components, we performed ablation experiments by individually removing the feature graph, the spatial graph, the DGI objective, and the spatial regularization term (Tables S7–S11). For each ablation setting, the remaining training and downstream clustering procedures were kept unchanged. We further compared the integrated SpaHDSRL model with single-modality controls on real datasets, with quantitative results summarized in Table S12. We also performed sensitivity analyses for the aggregation neighborhood size, downstream KNN graph size, and spatial regularization strength, and clustering number. Detailed settings and results are provided in Fig. S1 and Tables S13–S16.

## Results

### Overview of SpaHDSRL

**Figure 1:**
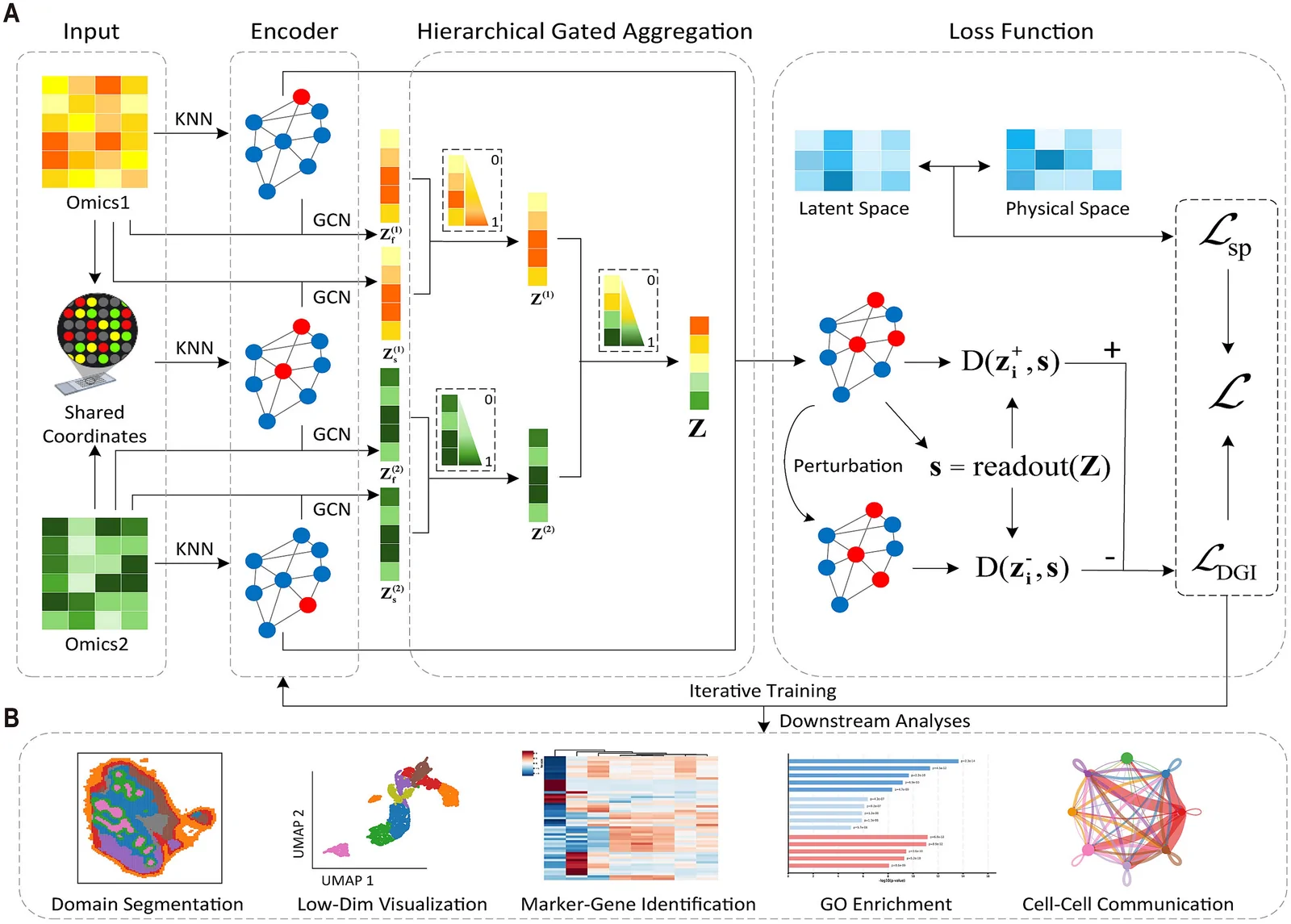
The overall framework of SpaHDSRL. (A) SpaHDSRL integrates spatial multi-omics input data by separately encoding spatial and molecular features from each modality using GCNs, followed by a hierarchical gated aggregation module to produce unified low-dimensional embedding. A combined optimization objective of DGI-based contrastive learning and spatial regularization preserves discriminative latent structures while maintaining spatial consistency. (B) The resulting integrated multi-omics embedding supports diverse downstream analyses, including spatial domain segmentation, low-dimensional visualization, marker gene identification, GO enrichment analysis, and cell–cell communication inference.

The goal of spatial multi-omics integration is to learn a unified latent representation that effectively combines information from different modalities. To this end, we propose SpaHDSRL, a graph-based framework that jointly models spatial adjacency and molecular similarity to capture complementary information across modalities. As illustrated in Fig. 1A, the SpaHDSRL framework consists of four modules: [1] Input module: SpaHDSRL takes modality-specific omics profiles and shared spatial coordinates as input. The spatial coordinates are used to construct a shared spatial adjacency graph that characterizes tissue organization, whereas the omics profiles of each modality are used to build modality-specific feature-similarity graphs that capture molecular relationships. [2] Encoder module: GCNs are applied separately to the shared spatial graph and the modality-specific feature-similarity graphs, producing spatial-context and feature-similarity embeddings for each modality. In this way, both the spatial organization of spots and the molecular similarity within each omics layer are retained in the encoded representations. [3] Fusion module: A hierarchical gated aggregation strategy adaptively integrates the spatial and feature embeddings within each modality and further performs cross-modality aggregation guided by inter-modality similarity to generate a unified latent representation. Through adaptive gating, spatial, feature-level, and cross-modal information are balanced in a data-driven manner. [4] Loss function module: The model is optimized using a DGI objective together with a spatial regularization term. The DGI objective improves the informativeness of the learned representation in an unsupervised manner, while the spatial regularization term encourages the latent embedding to remain consistent with the physical tissue structure. The ablation experiments further confirmed the contribution of the dual-graph design, hierarchical fusion strategy, DGI objective, and spatial regularization, with the full SpaHDSRL model generally achieving the best performance across datasets (Tables S7–S11).

The integrated embedding learned by SpaHDSRL is then used for downstream analyses, as shown in Fig. 1B, including spatial domain segmentation, low-dimensional visualization, marker-gene identification, functional enrichment analysis and cell–cell communication inference. These analyses enable SpaHDSRL to characterize spatial tissue organization and uncover biologically meaningful patterns across modalities.

### Benchmarking SpaHDSRL and existing methods on simulated datasets

We conducted benchmarking experiments on two simulated spatial multi-omics datasets with ground-truth labels. Five integration methods (pyWNN, Multigrate, COSMOS, SpatialGlue, and SpaHDSRL) were applied to these datasets. Given the availability of ground truth, we adopted six supervised clustering metrics for evaluation, including Adjusted Rand Index (ARI), Normalized Mutual Information (NMI), Fowlkes Mallows Index (FMI), Homogeneity, Completeness, and V-measure. Detailed descriptions of these metrics are provided in Supplementary S2. Based on these metrics, we systematically assessed the accuracy of spatial domain identification across both simulated datasets.

**Figure 2:**
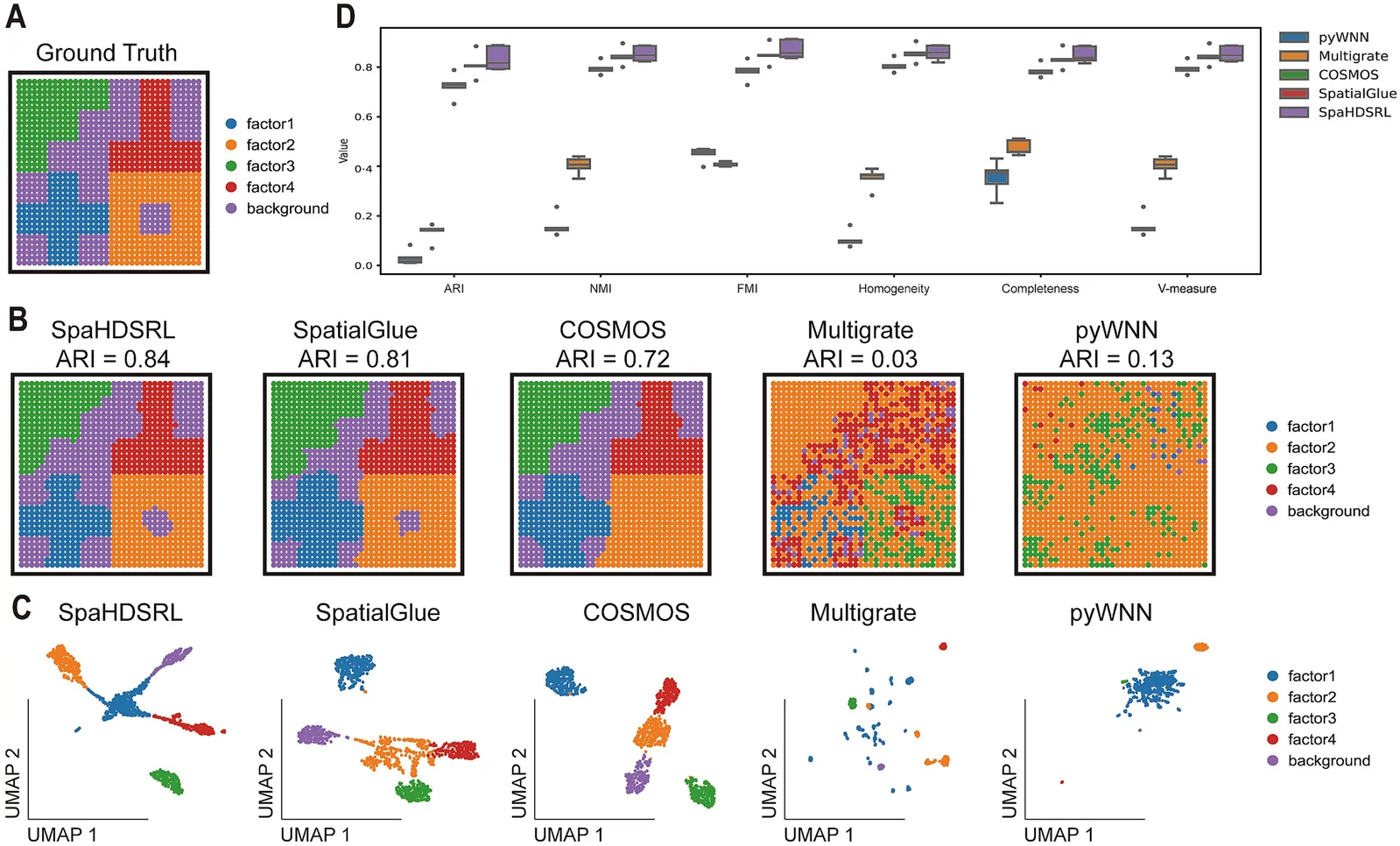
Performance evaluation of SpaHDSRL against state-of-the-art methods on the first simulated dataset. (A) Ground-truth spatial domain labels of the first simulated dataset. (B) Spatial domain segmentation results produced by five integration methods, including pyWNN, Multigrate, COSMOS, SpatialGlue, and SpaHDSRL. (C) UMAP visualization of the integrated embeddings generated by the five methods. (D) Quantitative evaluation of clustering performance for the five methods based on five independent runs, using ARI, NMI, FMI, Homogeneity, Completeness, and V-measure.

For the first simulated dataset, which contains both unique and complementary information across modalities [5], we compared SpaHDSRL with other competing methods in terms of spatial domain identification accuracy (Fig. 2). As shown in Fig. 2B, SpaHDSRL accurately recovers the underlying spatial structures and preserves clear region boundaries that closely match the ground truth. In contrast, pyWNN and Multigrate exhibit substantial noise and fail to form coherent spatial domains, while SpatialGlue partially captures the global structure but shows blurred boundaries in several regions. These results indicate that SpaHDSRL can exploit spatial information more effectively to reconstruct biologically meaningful tissue organization. In addition, we further visualized the spatial domain identification results in the UMAP space (Fig. 2C). The representations learned by SpaHDSRL form well-separated and compact clusters with clear distinctions among different spatial domains. By comparison, pyWNN and Multigrate produce highly mixed or fragmented clusters. Although SpatialGlue and COSMOS achieve a certain degree of separation, their clusters are less compact than those produced by SpaHDSRL, indicating relatively weaker representation quality. To provide a more intuitive comparison of spatial domain identification performance, we evaluated SpaHDSRL and the competing methods using multiple clustering metrics and visualized the results using boxplots in Fig. 2D. By performing five independent runs with different random seeds, SpaHDSRL consistently achieves the highest average scores across all metrics, with 0.835 (ARI), 0.854 (NMI), 0.872 (FMI), 0.858 (Homogeneity), 0.850 (Completeness), and 0.854 (V-measure). These results further demonstrate the superior accuracy and robustness of SpaHDSRL in spatial domain identification. The detailed results for all methods are provided in Table S2.

**Figure 3:**
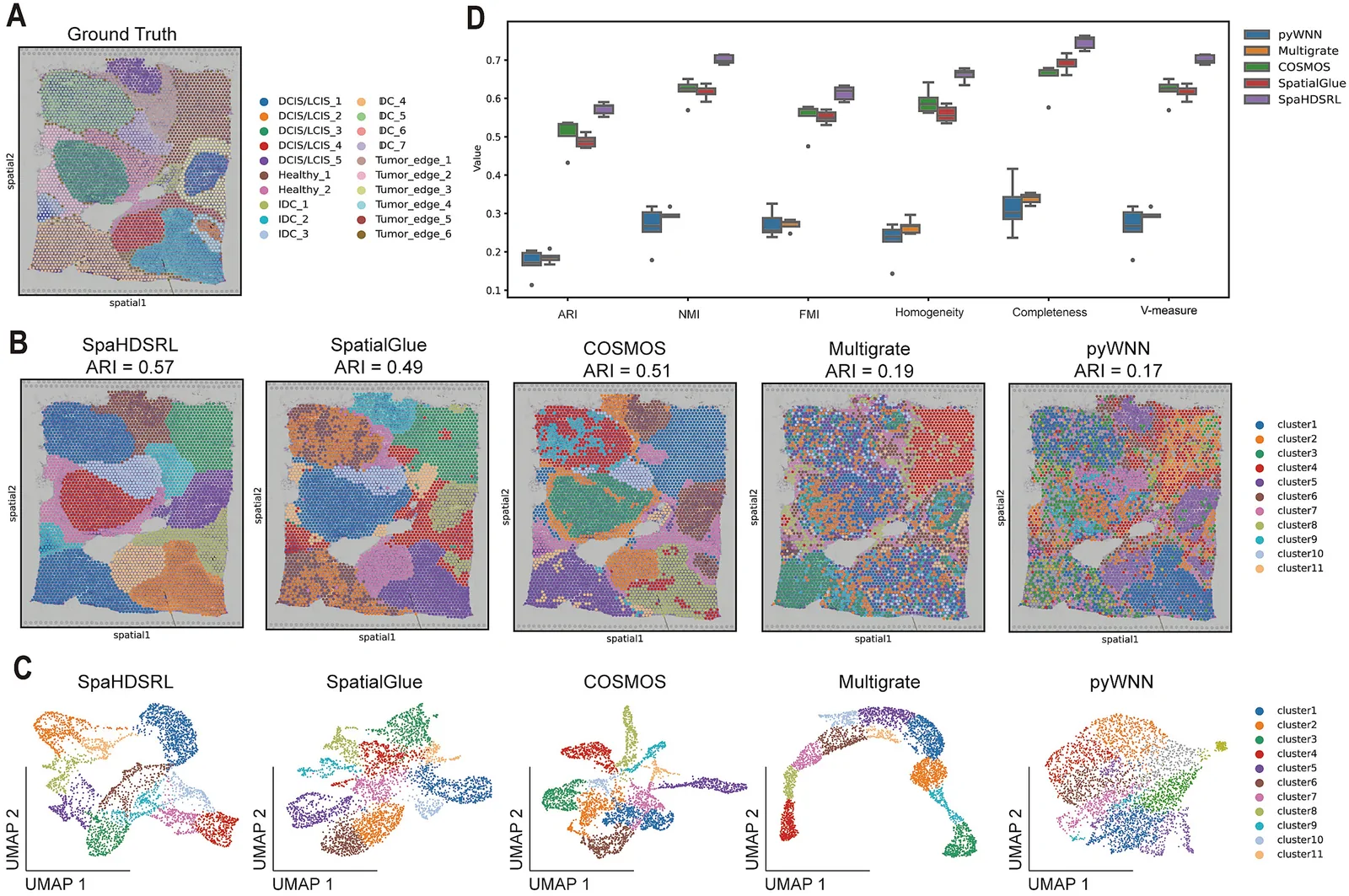
Performance evaluation of SpaHDSRL against state-of-the-art methods on the second simulated dataset. (A) Ground-truth spatial domain labels of the second simulated dataset. (B) Spatial domain segmentation results produced by five integration methods. (C) UMAP visualization of the integrated embeddings generated by the five methods. (D) Quantitative evaluation of clustering performance for the five methods based on five independent runs, using ARI, NMI, FMI, Homogeneity, Completeness, and V-measure.

For the second dataset, we constructed a simulated multi-omics data based on the human breast cancer Visium transcriptomics data. Specifically, we shuffled the correspondence between cells from IDC 5/IDC 4 and IDC 5/IDC 2, and further added Gaussian noise to the expression profiles (see Methods). This setting introduces both cross-modality inconsistency and measurement noise, providing a more challenging and realistic scenario for evaluation. We compared SpaHDSRL with other competing methods on this simulated dataset (Fig. 3). As shown in Fig. 3B, SpaHDSRL maintains coherent spatial regions and produces smoother transitions between neighboring areas under this noisy setting. In contrast, pyWNN and Multigrate exhibit severe fragmentation and fail to preserve meaningful spatial continuity. Notably, the regions identified by SpaHDSRL are well aligned with major histological structures, such as IDC and tumor-edge areas, suggesting improved biological consistency and stronger robustness to perturbations. The UMAP visualization (Fig. 3C) shows that the embedding learned by SpaHDSRL form more compact and well-separated clusters compared with other competing methods. In particular, regions corresponding to ductal carcinoma *in situ* / lobular carcinoma *in situ* (DCIS/LCIS) and tumor cells are more clearly separated in the UMAP space, whereas pyWNN and SpatialGlue show more mixed cluster distributions. Furthermore, the boxplots in Fig. 3D provide a quantitative comparison of spatial domain identification performance across different methods. Across five independent runs with different random seeds, SpaHDSRL consistently achieves the best average performance, with scores of 0.573 (ARI), 0.701 (NMI), 0.614 (FMI), 0.661 (Homogeneity), 0.746 (Completeness), and 0.701 (V-measure). In addition, SpaHDSRL shows smaller variance than COSMOS and pyWNN across most metrics, indicating improved stability and robustness to random initialization. The detailed results for all methods are provided in Table S3.

Across two simulated datasets, SpaHDSRL consistently outperforms other competing methods in terms of spatial domain identification accuracy, latent representation quality, and quantitative clustering performance. These results demonstrate the ability of SpaHDSRL to produce more accurate and robust spatial domain segmentation under both complementary multi-modal information and noisy cross-modality inconsistency settings.

### SpaHDSRL characterizes spatial developmental molecular features in the E10 mouse embryonic brain

Furthermore, we evaluated SpaHDSRL’s utility on a real spatial multi-omics dataset derived from the E10 mouse embryonic brain, with the aim of assessing its ability to resolve spatial domains in biologically complex tissues. Five integration methods were applied for spatial domain segmentation, and the tissue was uniformly partitioned into 10 regions for comparison.

**Figure 4:**
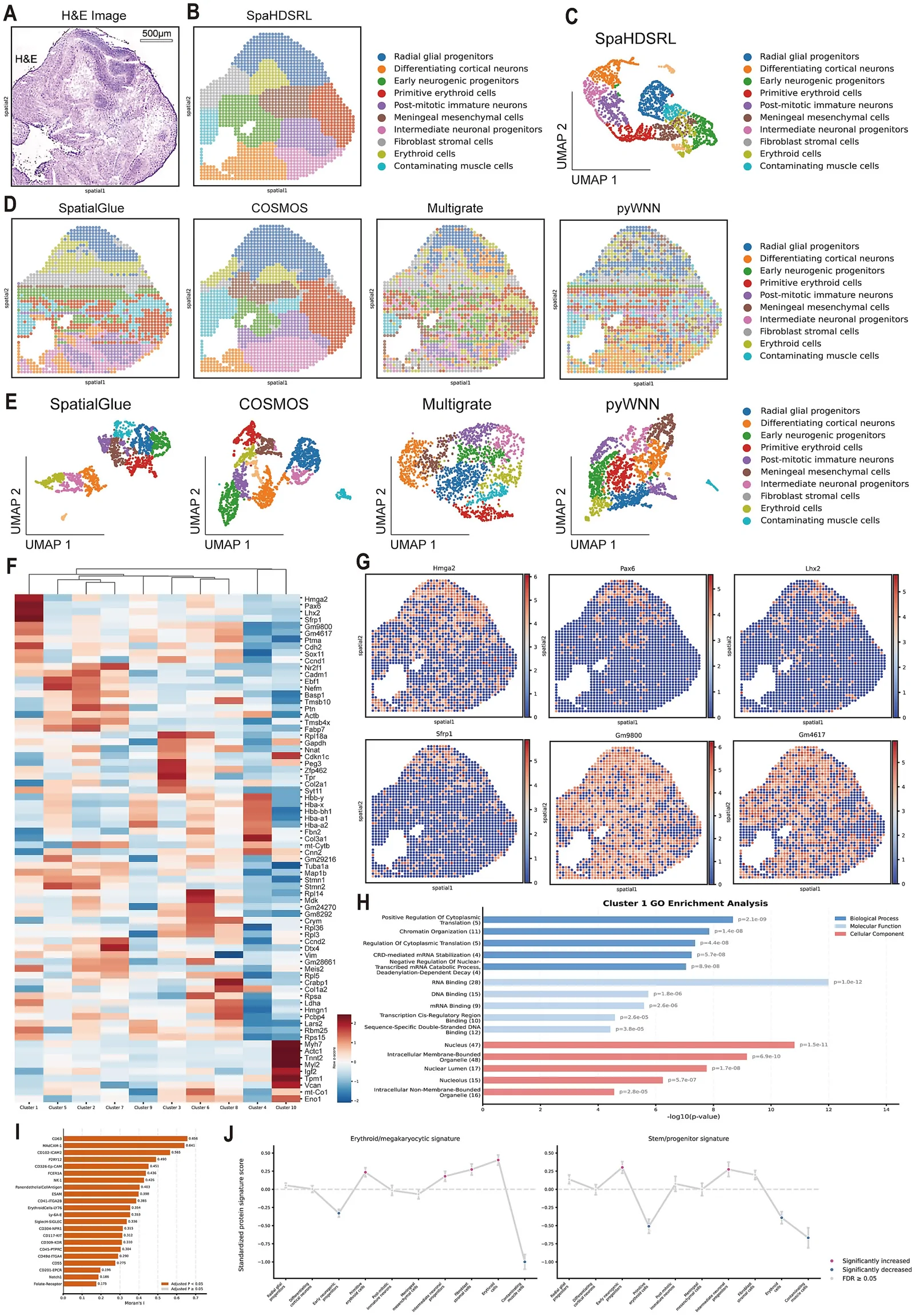
SpaHDSRL analysis on spatially resolved E10 mouse embryonic brain dataset. (A) Hematoxylin and eosin- stained image of the tissue section. (B) Spatial domain segmentation result generated by SpaHDSRL. (C) UMAP visualization of the integrated embedding learned by SpaHDSRL. (D) Spatial domain segmentation results produced by the four existing methods (SpatialGlue, COSMOS, Multigrate and pyWNN). (E) UMAP visualizations of the integrated embeddings generated by the four methods. (F) Heatmap showing the expression profiles of differentially expressed genes. (G) Spatial expression heatmaps of the top six differentially expressed genes in Cluster 1. (H) GO enrichment analysis revealing the biological functions associated with Cluster 1. (I) Moran’s I scores of all 22 protein ranked by spatial autocorrelation. (J) Domain-level protein signature scores of erythroid/megakaryocytic and stem/progenitor programs.

The spatial clustering results revealed substantial differences among methods in spatial continuity and boundary resolution. Guided by subsequent cell-type annotation, domains identified by SpaHDSRL corresponded closely to major developmental populations in the embryonic brain (Fig. 4B). For instance, radial glial progenitors displayed a clear laminar relationship with adjacent progenitor-associated regions, whereas differentiating cortical and post-mitotic immature neurons formed neighboring yet distinguishable domains. In contrast, Multigrate and pyWNN produced more local mixing and less well-defined boundaries, while SpatialGlue and COSMOS partially recovered the global hierarchy but showed broader transition zones. Consistently, the UMAP visualization showed that the SpaHDSRL embedding achieved clearer separation of most spatial domains in the low-dimensional space (Fig. 4C). Quantitative evaluation further supported these observations. SpaHDSRL achieved the best performance across all five spatial evaluation metrics, with a Neighborhood Label Agreement of 0.922, Domain Compactness of 0.846, Largest Connected Component Ratio of 1.000, Moran’s I of 0.913, and Geary’s C of 0.087 (Table S4).

To assess the biological relevance of the segmented domains, we performed differential expression analysis based on the SpaHDSRL clustering results (Fig. 4F). Each cluster exhibited a distinct transcriptional signature, with marker genes preferentially enriched within their corresponding spatial domains. We focused on Cluster 1, which was annotated as radial glial progenitors (RGPs). This cluster showed strong expression of canonical neural progenitor markers, including Hmga2, Pax6, Lhx2, and Sfrp1. Among these, Pax6 is a key transcription factor for neural stem cell proliferation, multipotency, and neurogenesis [24], specifically expressed in radial glial cells to control their differentiation and neurogenic capacity [25]. Lhx2 functions as a critical transcriptional regulator of neural differentiation and cortical patterning, partly through Pax6-related networks [26]. Hmga2, an architectural chromatin-binding protein, is highly expressed in fetal neural stem cells and promotes stem cell self-renewal and proliferation during early development [27]. Sfrp1, as a secreted antagonist of the Wnt signaling pathway, regulates neural progenitor activity by modulating Wnt signaling gradients and has been shown to maintain progenitor quiescence and control activation of neural stem cells [28]. These markers were strongly spatially restricted and highly expressed in Cluster 1 compared to other clusters (Fig. 4F), supporting the interpretation that this cluster represents a progenitor-enriched region with active developmental potential.

To further characterize the functional properties of this cluster, we performed GO enrichment analysis (Fig. 4H), which summarizes the enriched terms according to their statistical significance and associated gene counts. The enriched terms included RNA binding, DNA binding, mRNA binding, and chromatin organization, which are central to transcriptional and post-transcriptional regulation. These processes are essential for neural progenitor cells, as they require dynamic control of gene expression to balance self-renewal and differentiation [29]. For instance, RNA-binding proteins regulate mRNA stability and translation, thereby influencing neural stem cell fate decisions [30]. Additionally, enrichment of nuclear-related terms such as nucleus and chromatin organization suggests active chromatin remodeling and transcriptional activity, which are hallmarks of proliferative radial glial cells [24]. The enrichment of regulation of cytoplasmic translation further indicates elevated protein synthesis required to support rapid proliferation and differentiation during early brain development.

In addition, all 22 measured protein features exhibited significant positive spatial autocorrelation, with Moran’s I values ranging from 0.175 to 0.656, indicating that the protein modality retained spatially organized signals across the tissue (Fig. 4I). Domain-level protein signature analysis further showed that the erythroid/megakaryocytic signature was elevated in erythroid-related domains, consistent with the emergence of primitive hematopoietic and erythroid/megakaryocytic lineages during early mouse embryogenesis. The stem/progenitor signature showed a distinct pattern, which increased in neurogenic progenitor-associated domains and reduced in erythroid or contaminating muscle cell domains (Fig. 4J). These protein-level patterns were consistent with the transcriptomic marker-gene and GO enrichment analyses, while also providing an additional modality-specific view of lineage and cell-state differences among the inferred spatial domains.

Collectively, these results demonstrate that the spatial domains identified by SpaHDSRL are not only spatially well organized, but also consistent with the anatomical structure of the tissue in terms of biological function.

### SpaHDSRL resolves transcriptome-chromatin coordinated spatial patterning during E13 mouse embryogenesis

To further assess the generalizability of SpaHDSRL across distinct biological contexts, we extended our analysis to a spatial E13 mouse embryo RNA-ATAC dataset. We applied five methods to it and compared their abilities to delineate spatial domains in the developing embryo.

**Figure 5:**
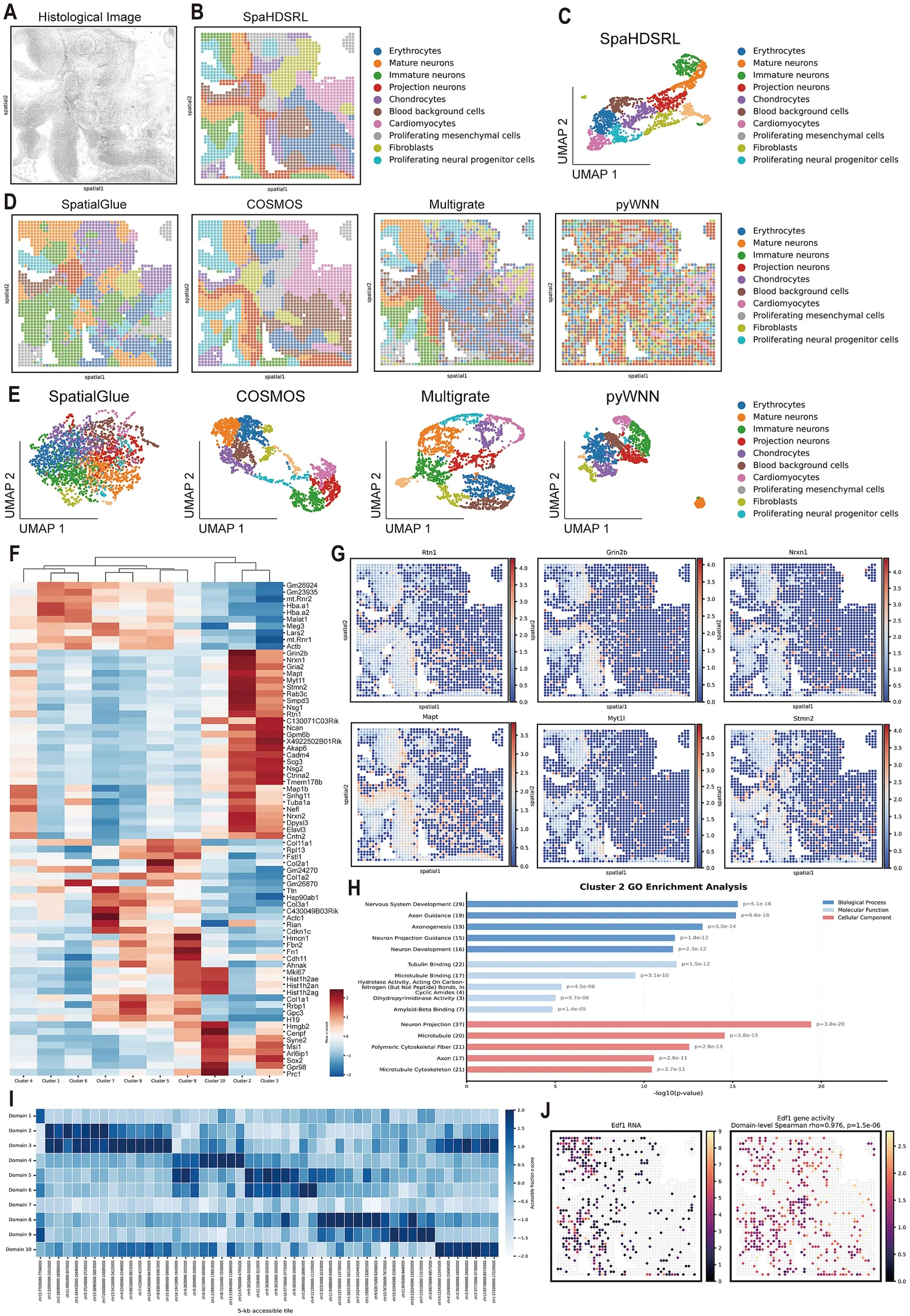
SpaHDSRL analysis on spatially resolved E13 mouse embryo dataset. (A) Histological image of the tissue section. (B) Spatial domain segmentation result generated by SpaHDSRL. (C) UMAP visualization of the integrated embedding learned by SpaHDSRL. (D) Spatial domain segmentation results produced by the four existing methods (SpatialGlue, COSMOS, Multigrate and pyWNN). (E) UMAP visualizations of the integrated embeddings generated by the four methods. (F) Heatmap showing the expression profiles of differentially expressed genes. (G) Spatial expression heatmaps of the top six differentially expressed genes in Cluster 2. (H) GO enrichment analysis revealing the biological functions associated with Cluster 2. (I) Heatmap showing domain-level z-scored accessibility patterns of 5-kb accessible tiles. (J) Spatial visualization of Edf1 RNA expression and ATAC-derived gene activity, with domain-level Spearman correlation indicating concordant RNA-ATAC patterns.

The spatial partitioning results revealed clear differences in domain organization among methods (Fig. 5B, D). SpaHDSRL identified domains with stronger structural coherence, partitioning the tissue into relatively continuous spatial units while more naturally separating neural-associated regions from blood- and mesenchyme-associated regions. By contrast, Multigrate and pyWNN produced less distinct boundaries, with noticeable mixing among adjacent populations. SpatialGlue partially preserved the global spatial hierarchy, but several regions still showed broad transition zones. Consistent with spatial segmentation results, UMAP visualization (Fig. 5C) showed that the latent representation learned by SpaHDSRL achieved clearer separation of distinct spatial domains. In particular, domains associated with neural development and those related to blood or mesenchymal programs occupied more separated regions in the embedding space, whereas several clusters remained substantially overlapped in SpatialGlue. This improvement was also reflected in most spatial evaluation metrics, with SpaHDSRL achieving the best performance in Neighborhood Label Agreement, Largest Connected Component Ratio, Moran’s I, and Geary’s C, while COSMOS showed a slightly higher Domain Compactness score (Table S5).

We next examined the molecular characteristics of the segmented domains. Distinct transcriptional features were observed across spatial regions (Fig. 5F). In addition to domain-specific markers, several genes displayed relatively broad expression across multiple domains, suggesting shared basal transcriptional activity during embryonic development. For example, blood-associated genes such as Hba-a1, Hba-a2, and Hba-x were selectively enriched in specific clusters, consistent with erythroid- or blood-associated regions. By contrast, neural markers including Grin2b, Nrxn1, Mapt, and Stmn2 were concentrated in another subset of clusters, indicating neuronal differentiation and neural structural specialization. The accessibility heatmap also revealed clear domain-specific accessible tile patterns, with groups of 5-kb accessible regions preferentially enriched in distinct SpaHDSRL domains (Fig. 5I). We then compared RNA expression with ATAC-derived gene activity at the domain level (Fig. 5J). As representative examples, Edf1 and Kdm1a showed concordant spatial patterns between RNA expression and ATAC-derived gene activity, with strong domain-level correlations (Edf1: Spearman, $\rho=0.988,\ p=9.3\times 10^-8$; Kdm1a: Spearman $\rho=0.976,\ p=1.5\times 10^-6$). These results indicate that the SpaHDSRL-defined domains capture coordinated transcriptome-chromatin variation, with the ATAC modality providing regulatory context that complements RNA-based domain interpretation.

We further focused on Cluster 2, annotated as mature neurons. This cluster was characterized by high expression of Rtn1, Grin2b, Nrxn1, Mapt, Myt1l, and Stmn2 (Fig. 5G). Among these, Grin2b encodes the GluN2B subunit of the NMDA receptor and is involved in excitatory synaptic transmission and long-term synaptic plasticity, thereby contributing to neuronal connectivity and circuit formation [31]. Mapt and Stmn2 are associated with microtubule regulation and neurite extension [32, 33], while Myt1l has been implicated in neuronal differentiation and maintenance [34]. These features suggest that Cluster 2 represents a spatial region enriched for neurons undergoing active neurite growth and neural circuit assembly. GO enrichment analysis further supported this interpretation (Fig. 5H). Differentially expressed genes in Cluster 2 were significantly enriched in axon guidance, axonogenesis, and neuron projection development, which are core processes governing neural circuit formation. These processes coordinate axonal pathfinding and synaptic targeting during development. Additionally, enrichment of microtubule binding and cytoskeletal organization terms indicates active cytoskeletal remodeling, which is essential for axon elongation, intracellular transport, and neuronal structural plasticity. Such processes are hallmarks of neurons undergoing synaptic maturation and circuit assembly, where synaptic plasticity and structural remodeling occur simultaneously [35].

In conclusion, SpaHDSRL effectively integrates the paired modalities and delineates biologically meaningful spatial domains, with Cluster 2 specifically representing a neuron-enriched region associated with neurite outgrowth, synaptic organization, and cytoskeletal dynamics.

### SpaHDSRL reveals spatially organized immune heterogeneity in the mouse thymus

We then applied SpaHDSRL to a Stereo-CITE-seq mouse thymus dataset. As a central immune organ for T-cell development, differentiation and maturation, the thymus exhibits well-organized yet highly complex spatial architecture, making it suitable for evaluating SpaHDSRL in resolving functionally distinct regions and immune cell populations.

**Figure 6:**
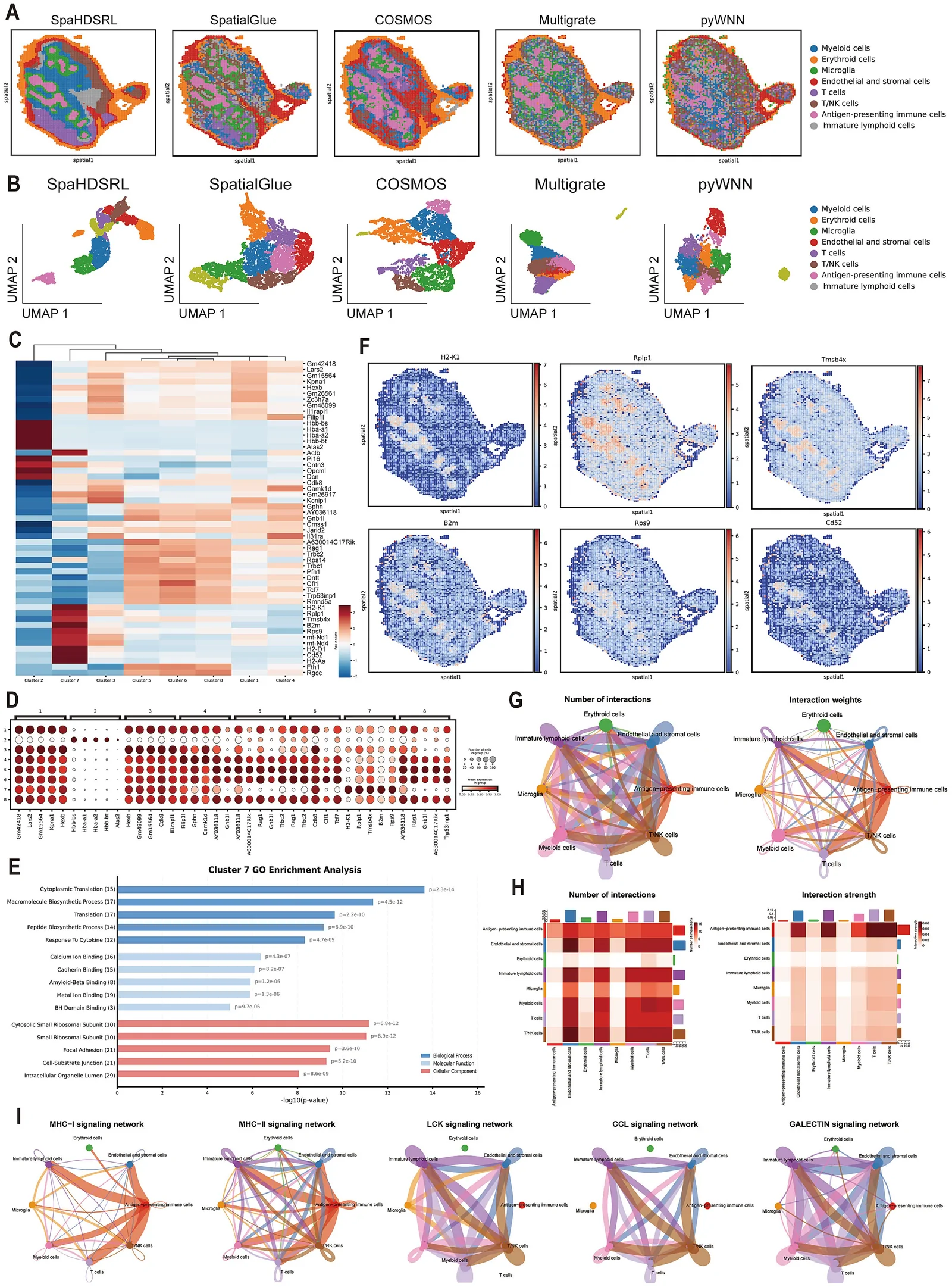
SpaHDSRL analysis on spatially resolved Stereo-CITE-seq mouse thymus dataset. (A) Spatial domain segmentation result generated by five integration methods (SpaHDSRL, SpatialGlue, COSMOS, Multigrate and pyWNN). (B) UMAP visualization of the integrated embedding learned by the five methods. (C) Heatmap showing the expression profiles of differentially expressed genes. (D) Dotplot visualization of differential expression patterns. (E) GO enrichment analysis revealing the biological functions associated with Cluster 7. (F) Spatial expression heatmaps of the top six differentially expressed genes in Cluster 7. (G) Circle plots depicting the intercellular communication network, with node sizes proportional to cell group sizes and edge thickness representing either the number of interactions (left) or interaction weights (right). (H) Heatmaps showing intercellular communication, displaying the number of interactions (left) and interaction strength (right). (I) Circle plots of the top five signaling pathways (MHC-I, MHC-II, LCK, CCL, and GALECTIN).

As shown in Fig. 6A, SpaHDSRL recovered coherent and biologically interpretable thymic regions, while the competing methods showed varying degrees of mixing or fragmentation. The UMAP visualization further showed that SpaHDSRL produced more compact and better-separated clusters, suggesting a more discriminative latent representation of the thymic microenvironment (Fig. 6B). Also, SpaHDSRL achieved the best scores across all five evaluation measures (Table S6). In particular, SpaHDSRL showed higher neighborhood consistency and spatial autocorrelation than the competing methods, with a Neighborhood Label Agreement of 0.776 and Moran’s I of 0.740, while also obtaining the lowest Geary’s C value of 0.260. These results indicate that the SpaHDSRL-derived thymic domains were more spatially coherent and consistent with local tissue organization.

We further characterized the segmented domains using cluster-specific differentially expressed genes. The marker heatmap revealed clear transcriptional differences across clusters, indicating that the SpaHDSRL-identified domains corresponded to biologically distinct cellular niches rather than only contiguous anatomical regions (Fig. 6C). Among them, Cluster 7 was annotated as an antigen-presenting-associated immune domain and selected for further analysis (Fig. 6D). This cluster showed elevated expression of H2-K1 and B2m, two key components of the MHC class I antigen-presentation machinery. In vertebrates, class I MHC molecules are formed by a polymorphic heavy chain together with β2-microglobulin, and this complex is essential for presenting intracellular peptides to CD8 T cells [36, 37]. Cluster 7 also showed increased expression of Cd52, a GPI-anchored glycoprotein broadly expressed in mature lymphocytes and also detected in monocytes and dendritic cells, making it a well-established immune-lineage marker rather than a generic tissue gene [38, 39]. Thus, the combined elevation of H2-K1, B2m, and Cd52 supports the interpretation that this domain corresponds to an immune-active thymic niche with antigen-presentation potential. In addition, the relatively high expression of Rplp1 and Rps9 suggests enhanced ribosome-associated biosynthetic activity. Ribosomal proteins are structural and functional components of the translation machinery, and elevated expression of ribosomal-protein genes is commonly associated with active protein synthesis. Notably, Rplp1 has been shown to contribute to translational control required for embryonic development and cell proliferation [40], suggesting that Cluster 7 is in a metabolically and functionally active state rather than a quiescent immune compartment.

This interpretation was supported by GO enrichment analysis (Fig. 6E). The enriched biological-process terms, including cytoplasmic translation, translation, and macromolecule/peptide biosynthetic process, are consistent with elevated protein synthesis. Terms related to cytokine response suggest sensitivity to immune signaling cues, which is relevant to cytokine-mediated regulation of thymic immune-cell differentiation and selection. In addition, cellular-component terms such as focal adhesion and cell-substrate junction indicate strengthened adhesion interfaces with the local microenvironment, consistent with the role of thymic antigen-presenting and non-T-lineage immune cells in engaging developing thymocytes during selection and tolerance induction [41, 42]. Collectively, these results suggest that Cluster 7 represents a functionally active immune-associated niche marked by enhanced MHC-I-related antigen presentation, translational activity, cytokine responsiveness, and adhesion-linked interactions with the surrounding thymic tissue.

We then used this dataset as a representative case for CellChat analysis to investigate ligand-receptor-mediated communication patterns associated with SpaHDSRL-identified spatial domains [23]. At the global level (Fig. 6G and H), the communication network revealed extensive crosstalk among major thymic populations. In terms of interaction number, endothelial and stromal cells, immature lymphoid cells, myeloid cells, and T/NK cells formed dense connections with multiple populations, consistent with the coordinated roles of stromal and hematopoietic compartments in thymocyte migration, differentiation, and selection [42]. Stromal-derived signals are known to guide thymocyte trafficking and developmental progression, whereas thymic antigen-presenting and innate immune cells contribute to selection and tolerance induction [43]. In terms of interaction strength, antigen-presenting immune cells emerged as a prominent outgoing signaling source, strongly communicating with endothelial/stromal cells, immature lymphoid cells, T cells, and T/NK cells, in line with the role of thymic antigen-presenting cells in peptide–MHC-mediated selection and tolerance induction [41].

Pathway-level analysis further highlighted distinct signaling patterns among thymic populations (Fig. 6I). MHC-I and MHC-II signaling were dominated by antigen-presenting immune cells, supporting their role in self-antigens presentation during thymic selection and tolerance induction [44]. LCK signaling was mainly associated with immature lymphoid, T, and T/NK populations, consistent with its function as a proximal kinase in T-cell receptor signaling and thymic selection [45]. Similarly, CCL signaling was enriched among immature lymphoid, myeloid, T/TNK, and endothelial/stromal populations, reflecting the importance of thymic chemokines in thymocyte seeding, migration, and spatial positioning within stromal niches [43, 46]. GALECTIN signaling indicated broad immune-stromal communication. Galectin-9 and related galectin pathways are known to regulate immune-cell activation, apoptosis, and homeostasis, and in the thymus LGALS9-associated signaling has been implicated in thymocyte apoptosis and stromal-immune communication [47, 48].

Overall, these results indicate that the spatial domains identified by SpaHDSRL can serve as an effective organizational basis for downstream biological analyses, enabling the exploration of intercellular communication patterns in the thymic microenvironment.

## Conclusion

We developed a hierarchical dual-graph self-supervised representation learning framework for spatial multi-omics integration, termed SpaHDSRL. Specifically, SpaHDSRL models a shared spatial graph together with modality-specific feature graphs, enabling the framework to preserve both spatial neighborhood structure and molecular similarity. On this basis, SpaHDSRL adopts a hierarchical fusion strategy that first integrates spatial and molecular representations within each modality through spot-specific gates and then performs gated cross-modality aggregation to derive a unified latent representation. Together with DGI-based optimization and spatial regularization, this design allows SpaHDSRL to learn spatially coherent and biologically informative representations. Finally, performance evaluation on simulated and real datasets showed that SpaHDSRL achieved improved performance over several representative methods in the evaluated datasets, producing spatial domains with better spatial coherence and biological interpretability, while supporting downstream analyses such as marker gene characterization, functional enrichment, second-modality-associated analysis, and cell–cell communication analysis.

Despite these promising results, several practical considerations should be noted. First, SpaHDSRL is primarily designed for paired spatial multi-omics data in which different modalities are measured at the same spatial locations, and is therefore not applicable to the unpaired datasets. Second, given its directional cross-modality fusion mechanism, the framework is currently most suitable when a relatively information-rich modality, such as RNA, is used as the reference modality and the other modality provides complementary information. Consequently, SpaHDSRL may be sensitive to the ordering of the input modalities, particularly in real datasets. Future work could explore symmetric or bidirectional fusion strategies to reduce this modality-order dependence. Additionally, SpaHDSRL does not explicitly model more complex biological dependencies, such as long-range spatial interactions, dynamic developmental trajectories, or higher-order relationships among cells and tissue regions. Extending the framework to capture multi-scale and dynamic biological organization may therefore further improve its ability to characterize complex tissues. In addition, incorporating a broader range of downstream analyses may further expand the applicability of SpaHDSRL and enhance its utility in spatial multi-omics studies.

**Key Points**

- We present SpaHDSRL, a hierarchical dual-graph self-supervised representation learning framework for spatial multi-omics integration, designed to learn unified representation from paired spatially resolved omics modalities.
- SpaHDSRL introduces dual-graph modeling to effectively preserve local spatial dependencies and molecular similarity. Its hierarchical gated fusion strategy adaptively integrates spatial- and feature-level information within each modality and complementary information across modalities, enabling the model to learn coherent and biologically informative representations.
- Evaluations on simulated and real spatial multi-omics datasets show that SpaHDSRL achieves improved performance compared with several representative methods in the evaluated datasets, while supporting downstream biological analyses such as marker gene characterization, functional enrichment, second-modality-associated analysis, and cell–cell communication inference.

## Supplementary Material

Supplementary_material_bbag517

## Data Availability

All data used in this study are publicly available. The two simulated datasets have been released at github (https://github.com/Lisa62103/SpaHDSRL/tree/main/Data). The DBiT-seq E10 mouse embryonic brain data is available at figshare database (https://figshare.com/articles/dataset/Spatial_genomics_datasets/21623148/5). The E13 mouse embryo data is obtained from the Gene Expression Omnibus (GEO) repository (accession no GSM6801813), and the Stereo-CITE-seq mouse thymus data is obtained from BGI. The source code for SpaHDSRL, along with the tutorial notebooks for reproducing the results in this paper, is available at GitHub (https://github.com/Lisa62103/SpaHDSRL).
