# Supplementary material for: SpaHDSRL: hierarchical dual-graph self-supervised representation learning for integrating spatially resolved multi-omics data
Source: Brief Bioinform. 2026 Sep 18;27(5):bbag517. doi: 10.1093/bib/bbag517 (PMC13587101; doi:10.1093/bib/bbag517)
Supplement: Supplementary file 1 [file supplementary_material_bbag517.zip › Supplementary_material_captions.docx]

**Supplementary material**

Supplementary Data 1. Supplementary Illustration.

Supplementary Data 2. Definition of supervised clustering evaluation metrics.

Supplementary Data 3. Definition of annotation-independent spatial evaluation metrics

Supplementary Data 4. Table S1. Details of real datasets used in experiments.

Supplementary Data 5. Table S2. Quantitative comparison of SpaHDSRL with competing methods on the first simulated dataset across six evaluation metrics.

Supplementary Data 6. Table S3. Quantitative comparison of SpaHDSRL with competing methods on the second simulated dataset across six evaluation metrics.

Supplementary Data 7. Table S4. Quantitative comparison of SpaHDSRL with competing methods on the E10 mouse embryonic brain dataset across five evaluation metrics.

Supplementary Data 8. Table S5. Quantitative comparison of SpaHDSRL with competing methods on the E13 mouse embryo dataset across five evaluation metrics.

Supplementary Data 9. Table S6. Quantitative comparison of SpaHDSRL with competing methods on the Stereo-CITE-seq mouse thymus dataset across five evaluation metrics.

Supplementary Data 10. Table S7. Ablation study of SpaHDSRL on the first simulated dataset.

Supplementary Data 11. Table S8. Ablation study of SpaHDSRL on the second simulated dataset.

Supplementary Data 12. Table S9. Ablation study of SpaHDSRL on the E10 mouse embryonic brain dataset.

Supplementary Data 13. Table S10. Ablation study of SpaHDSRL on the E13 mouse embryo dataset.

Supplementary Data 14. Table S11. Ablation study of SpaHDSRL on the Stereo-CITE-seq mouse thymus dataset.

Supplementary Data 15. Table S12. Quantitative comparison of single-modality controls and integrated SpaHDSRL on real datasets.

Supplementary Data 16. Table S13. Sensitivity analysis of KNN neighborhood size in cross-modal neighbor aggregation on the first simulated dataset.

Supplementary Data 17. Table S14. Sensitivity analysis of downstream KNN graph size in clustering performance on the first simulated dataset.

Supplementary Data 18. Table S15. Sensitivity analysis of the spatial regularization strength on the first simulated dataset.

Supplementary Data 19. Table S16. Sensitivity analysis of the clustering number on the E10 mouse embryonic brain dataset.

Supplementary Data 20. Table S17. Effect of modality-order swapping on the first simulated dataset.

Supplementary Data 21. Table S18. Effect of modality-order swapping on the E10 mouse embryonic brain dataset.

Supplementary Data 22. Figure S1. Sensitivity analyses of key SpaHDSRL parameters and visualization of learned gating weights on the first simulated dataset. (A) Spatial domain results obtained under different aggregation neighborhood sizes k. (B, C) Similarity distribution of selected top-k neighbors under different aggregation k values. (D) Clustering performance under different aggregation k values. (E) Spatial domain results obtained using different KNN graph sizes in the downstream Leiden clustering. (F) Clustering performance under different downstream Leiden KNN settings. (G) Spatial domain results under different spatial regularization strength $\lambda$ values. (H) Clustering performance under different spatial regularization strength $\lambda$ values. (I) Spatial distributions of learned intra-modality spatial-graph weights for each modality. (J) Spatial distributions of learned cross-modality gating weights for each modality.

Supplementary Data 23. Figure S2. Modality-order analysis and ablation studies of SpaHDSRL on simulated datasets. (A) Ground-truth spatial domains of the first simulated dataset. (B) Spatial clustering and UMAP visualization under the modality-swapped setting, where ADT was used as the first modality. (C) Spatial domain results of the full SpaHDSRL model and ablation variants on the first simulated dataset. (D) Quantitative comparison of the full model and ablation variants on the first simulated dataset. (E) Ground-truth spatial domains of the second simulated dataset. (F) Spatial domain results of the full model and ablation variants on the second simulated dataset. (G) Quantitative comparison of the full model and ablation variants on the second simulated dataset.

Supplementary Data 24. Figure S3. Modality-order analysis, single-modality controls, ablation studies, downstream analyses, and sensitivity analysis of SpaHDSRL on the E10 mouse embryonic brain dataset. (A) Spatial clustering and UMAP visualization under the modality-swapped setting, where protein was used as the first modality. (B, C) Spatial clustering and UMAP visualizations of the RNA-only and protein-only control models. (D) Spatial domain results of the full SpaHDSRL model and ablation variants. (E) Quantitative comparison of the full model and ablation variants across five spatial evaluation metrics. (F) Heatmap of the domain-level expression patterns of all 22 proteins. (G) Dotplot of the top five differentially expressed genes for each domain. (H) Spatial domain results obtained with different target clustering numbers. (I) Clustering performance under diiferent target clustering number.

Supplementary Data 25. Figure S4. Spatial expression heatmaps of all 22 proteins in the E10 mouse embryonic brain dataset.

Supplementary Data 26. Figure S5. Single-modality controls, ablation studies, and downstream analyses of SpaHDSRL on the E13 mouse embryo dataset. (A, B) Spatial clustering and UMAP visualizations of the RNA-only and ATAC-only control models. (C) Spatial domain results of the full SpaHDSRL model and ablation variants. (D) Quantitative comparison of the full model and ablation variants across five spatial evaluation metrics (E) Spatial distributions of representative accessible tiles across the tissue. (F) Moran’s I scores of the top ten spatially autocorrelated accessible tiles. (G) Spatial visualization of Kdm1a RNA expression and ATAC-derived gene activity. (H) Dotplot of the top five differentially expressed genes for each domain.

Supplementary Data 27. Figure S6. Single-modality controls, ablation studies, and downstream analyses of SpaHDSRL on the Stereo-CITE-seq mouse thymus dataset. (A, B) Spatial clustering and UMAP visualizations of the RNA-only and ADT-only control models. (C) Spatial domain results of the full SpaHDSRL model and ablation variants. (D) Quantitative comparison of the full model and ablation variants across five spatial evaluation metrics. (E) Spatial distributions of representative ADT features across the thymus tissue. (F) Moran’s I scores of all 33 measured ADT features ranked by spatial autocorrelation (G) Heatmap of the domain-level expression patterns of all 33 measured ADT features.
