# Supplementary material for: SpaHDSRL: hierarchical dual-graph self-supervised representation learning for integrating spatially resolved multi-omics data
Source: Brief Bioinform. 2026 Sep 18;27(5):bbag517. doi: 10.1093/bib/bbag517 (PMC13587101; doi:10.1093/bib/bbag517)
Supplement: Supplementary file 1 [file supplementary_material_bbag517.zip › Supplementary material.docx]

**Supplementary materials:**

**SpaHDSRL: Hierarchical Dual-Graph Self-Supervised Representation Learning for Integrating Spatially Resolved Multi-Omics Data**

**Table of Contents**

1. **Supplementary Illustration**

S1. Parameters of SpaHDSRL

S2. Definition of supervised clustering evaluation metrics

S3. Definition of annotation-independent spatial evaluation metrics

2. **Supplementary Tables**

Table S1. Details of real datasets used in experiments.

Table S2. Quantitative comparison of SpaHDSRL with competing methods on the first simulated dataset across six evaluation metrics.

Table S3. Quantitative comparison of SpaHDSRL with competing methods on the second simulated dataset across six evaluation metrics.

Table S4. Quantitative comparison of SpaHDSRL with competing methods on the E10 mouse embryonic brain dataset across five evaluation metrics.

Table S5. Quantitative comparison of SpaHDSRL with competing methods on the E13 mouse embryo dataset across five evaluation metrics.

Table S6. Quantitative comparison of SpaHDSRL with competing methods on the Stereo-CITE-seq mouse thymus dataset across five evaluation metrics.

Table S7. Ablation study of SpaHDSRL on the first simulated dataset.

Table S8. Ablation study of SpaHDSRL on the second simulated dataset.

Table S9. Ablation study of SpaHDSRL on the E10 mouse embryonic brain dataset.

Table S10. Ablation study of SpaHDSRL on the E13 mouse embryo dataset.

Table S11. Ablation study of SpaHDSRL on the Stereo-CITE-seq mouse thymus dataset.

Table S12. Quantitative comparison of single-modality controls and integrated SpaHDSRL on real datasets.

Table S13. Sensitivity analysis of KNN neighborhood size in cross-modal neighbor aggregation on the first simulated dataset.

Table S14. Sensitivity analysis of downstream KNN graph size in clustering performance on the first simulated dataset.

Table S15. Sensitivity analysis of the spatial regularization strength $\lambda$ on the first simulated dataset.

Table S16. Sensitivity analysis of the clustering number on the E10 mouse embryonic brain dataset.

Table S17. Effect of modality-order swapping on the first simulated dataset.

Table S18. Effect of modality-order swapping on the E10 mouse embryonic brain dataset.

3. **Supplementary Figures**

Figure S1. Sensitivity analyses of key SpaHDSRL parameters and visualization of learned gating weights on the first simulated dataset. (A) Spatial domain results obtained under different aggregation neighborhood sizes k. (B, C) Similarity distribution of selected top-k neighbors under different aggregation k values. (D) Clustering performance under different aggregation k values. (E) Spatial domain results obtained using different KNN graph sizes in the downstream Leiden clustering. (F) Clustering performance under different downstream Leiden KNN settings. (G) Spatial domain results under different spatial regularization strength $\lambda$ values. (H) Clustering performance under different spatial regularization strength $\lambda$ values. (I) Spatial distributions of learned intra-modality spatial-graph weights for each modality. (J) Spatial distributions of learned cross-modality gating weights for each modality.

Figure S2. Modality-order analysis and ablation studies of SpaHDSRL on simulated datasets. (A) Ground-truth spatial domains of the first simulated dataset. (B) Spatial clustering and UMAP visualization under the modality-swapped setting, where ADT was used as the first modality. (C) Spatial domain results of the full SpaHDSRL model and ablation variants on the first simulated dataset. (D) Quantitative comparison of the full model and ablation variants on the first simulated dataset. (E) Ground-truth spatial domains of the second simulated dataset. (F) Spatial domain results of the full model and ablation variants on the second simulated dataset. (G) Quantitative comparison of the full model and ablation variants on the second simulated dataset.

Figure S3. Modality-order analysis, single-modality controls, ablation studies, downstream analyses, and sensitivity analysis of SpaHDSRL on the E10 mouse embryonic brain dataset. (A) Spatial clustering and UMAP visualization under the modality-swapped setting, where protein was used as the first modality. (B, C) Spatial clustering and UMAP visualizations of the RNA-only and protein-only control models. (D) Spatial domain results of the full SpaHDSRL model and ablation variants. (E) Quantitative comparison of the full model and ablation variants across five spatial evaluation metrics. (F) Heatmap of the domain-level expression patterns of all 22 proteins. (G) Dotplot of the top five differentially expressed genes for each domain. (H) Spatial domain results obtained with different target clustering numbers. (I) Clustering performance under diiferent target clustering number.

Figure S4. Spatial expression heatmaps of all 22 proteins in the E10 mouse embryonic brain dataset.

Figure S5. Single-modality controls, ablation studies, and downstream analyses of SpaHDSRL on the E13 mouse embryo dataset. (A, B) Spatial clustering and UMAP visualizations of the RNA-only and ATAC-only control models. (C) Spatial domain results of the full SpaHDSRL model and ablation variants. (D) Quantitative comparison of the full model and ablation variants across five spatial evaluation metrics (E) Spatial distributions of representative accessible tiles across the tissue. (F) Moran’s I scores of the top ten spatially autocorrelated accessible tiles. (G) Spatial visualization of Kdm1a RNA expression and ATAC-derived gene activity. (H) Dotplot of the top five differentially expressed genes for each domain.

Figure S6. Single-modality controls, ablation studies, and downstream analyses of SpaHDSRL on the Stereo-CITE-seq mouse thymus dataset. (A, B) Spatial clustering and UMAP visualizations of the RNA-only and ADT-only control models. (C) Spatial domain results of the full SpaHDSRL model and ablation variants. (D) Quantitative comparison of the full model and ablation variants across five spatial evaluation metrics. (E) Spatial distributions of representative ADT features across the thymus tissue. (F) Moran’s I scores of all 33 measured ADT features ranked by spatial autocorrelation (G) Heatmap of the domain-level expression patterns of all 33 measured ADT features.

1. **Supplementary Illustration**

**S1. Parameters of SpaHDSRL**

Several parameters were kept constant across all analyses. Specifically, model training was performed using the Adam optimizer with a learning rate of 0.001, and the latent dimension was fixed at 50. To accelerate the computation of the spatial regularization term, regularization acceleration was enabled and the sampled edge subset size was set to ${10}^{6}$. The temperature parameter in the fusion module was fixed at 0.05.

Notably, two different neighborhood parameters were used in the framework. The parameter n_neighbors was used for constructing the spatial and feature graphs during preprocessing, whereas k_neighbors was used in the fusion module for top-k neighbor retrieval and weighted aggregation. n_neighbors was set to 5 for the simulated RNA-ADT dataset, Stereo-CITE-seq mouse thymus dataset and E13 mouse embryo dataset, 10 for the simulated RNA–protein dataset and DBiT-seq mouse embryo brain dataset. The parameter setting of k_neighbors is consistent with n_neighbors.

The spatial regularization strength was set to 0.07 for the simulated RNA-ADT dataset, 0.05 for the DBiT-seq mouse embryo brain dataset, 0.01 for the Stereo-CITE-seq mouse thymus dataset, 0.008 for the simulated RNA-protein dataset and E13 mouse embryo dataset.

The number of training epochs was set to 300 for the two simulated datasets and Stereo-CITE-seq mouse thymus dataset, 350 for the DBiT-seq mouse embryo brain dataset, and 500 for the E13 mouse embryo dataset.

**S2. Definition of supervised clustering evaluation metrics**

To quantitatively evaluate clustering performance on the simulated dataset, we adopted six supervised clustering metrics, including Adjusted Rand Index (ARI), Normalized Mutual Information (NMI), Fowlkes–Mallows Index (FMI), Homogeneity, Completeness and V-measure. These metrics compare the predicted clustering labels with the ground-truth labels, with higher values indicating better clustering performance.

Let $\mathcal{C}=\left\{ C_{1},\ldots,C_{r} \right\}$ denote the ground-truth classes and $\mathcal{K}=\left\{ K_{1},\ldots,K_{s} \right\}$ denote the predicted clusters. We define a contingency table $\mathbf{N}=\left( n_{ij} \right)$, where $n_{ij}=\left| C_{i}\cap K_{j} \right|$ represents the number of spots shared by the ground-truth class $C_{i}$ and the predicted cluster $K_{j}$. The row and column sums of the contingency table are denoted by $a_{i}=\sum_{j} n_{ij}$ and $b_{j}=\sum_{i} n_{ij}$, respectively, and the total number of spots is $n=\sum_{ij} n_{ij}$.

The Adjusted Rand Index (ARI) measures the agreement between two partitions based on pairwise spot assignments while adjusting for chance.

$\mathrm{ARI} =\frac{\sum_{ij} \left( \frac{n_{ij}}{2} \right)-\frac{\sum_{i} \left( \frac{a_{i}}{2} \right)\sum_{j} \left( \frac{b_{j}}{2} \right)}{\left( \frac{n}{2} \right)}}{\frac{1}{2}\left[ \sum_{i} \left( \frac{a_{i}}{2} \right)+\sum_{j} \left( \frac{b_{j}}{2} \right) \right]-\frac{\sum_{i} \left( \frac{a_{i}}{2} \right)\sum_{j} \left( \frac{b_{j}}{2} \right)}{\left( \frac{n}{2} \right)}}$.	(1)

The Normalized Mutual Information (NMI) measures the shared information between the ground-truth labels and predicted clusters.

$\mathrm{NMI} =\frac{2I\left( \mathcal{C};\mathcal{K} \right)}{H\left( \mathcal{C} \right)+H\left( \mathcal{K} \right)}$.	(2)

Where $I\left( \mathcal{C};\mathcal{K} \right)$ is the mutual information between $C$ and $K$, and $H\left( \cdot\right)$ denotes entropy.

The Fowlkes-Mallows Index (FMI) evaluates clustering accuracy based on pairwise precision and recall.

$\mathrm{FMI} =\frac{\mathrm{TP}}{\sqrt{\left( \mathrm{TP}+\mathrm{FP} \right)\left( \mathrm{TP}+\mathrm{FN} \right)}}$.	(3)

Where TP is the number of spot pairs assigned to the same cluster and the same ground-truth class, FP is the number of pairs assigned to the same predicted cluster but different ground-truth classes, and FN is the number of pairs assigned to the same ground-truth class but different predicted clusters.

Homogeneity evaluates whether each predicted cluster contains spots from a single ground-truth class.

$h=1-\frac{H\left( \mathcal{C}\mid\mathcal{K} \right)}{H\left( \mathcal{C} \right)}$.	(4)

Completeness evaluates whether all spots from the same ground-truth class are assigned to the same predicted cluster.

$c=1-\frac{H\left( \mathcal{K}\mid\mathcal{C} \right)}{H\left( \mathcal{K} \right)}$.	(5)

The V-measure is the harmonic mean of Homogeneity and Completeness.

$V=\frac{2hc}{h+c}$.	(6)

Together, these metrics provide complementary assessments of clustering consistency, information agreement, pairwise classification accuracy and label purity between the inferred spatial domains and the ground-truth annotations.

**S3. Definition of annotation-independent spatial evaluation metrics**

To quantitatively evaluate clustering performance on real datasets, we adopted five annotation-independent spatial evaluation metrics, including Neighborhood Label Agreement, Domain Compactness, Largest Connected Component Ratio, Moran’s I, and Geary’s C. These metrics do not require ground-truth labels and evaluate the spatial organization of predicted domains from different perspectives.

Let $G=\left( \mathcal{V},\mathcal{\mathcal{E}} \right)$ denote the spatial neighbor graph, where $\mathcal{V}=\left\{ 1,\ldots,n \right\}$ represents all spots and $E$ represents spatial neighboring relationships. Let $c_{i}$ denote the predicted domain label of spot $i$, and let $\mathcal{K}=\left\{ K_{1},\ldots,K_{s} \right\}$ denote the set of predicted domains. The spatial coordinate of spot $i$ is denoted as $\mathbf{p}_{i}$. The spatial adjacency weight between spots $i$ and $j$ is denoted as $w_{ij}$, and $W=\sum_{ij} w_{ij}$.

The Neighborhood Label Agreement measures the local consistency of predicted domain labels among spatially adjacent spots.

$\mathrm{NLA} =\frac{\sum_{i} \sum_{j} w_{ij}\mathbf{1}\left( c_{i}=c_{j} \right)}{\sum_{i} \sum_{j} w_{ij}}$.	(7)

The Domain Compactness evaluates whether spots assigned to the same predicted domain are spatially concentrated.

$\mathrm{DC} =1-\frac{\sum_{k} \sum_{i\in K_{k}} \left\| \mathbf{p}_{i}-\boldsymbol{\mu}_{k} \right\|_{2}}{\sum_{i} \left\| \mathbf{p}_{i}-\boldsymbol{\mu}_{0} \right\|_{2}}$,	(8)

Where $\boldsymbol{\mu}_{k}$ denotes the spatial centroid of domain $K_{k}$, and $\boldsymbol{\mu}_{0}$ denotes the centroid of all spots.

The Largest Connected Component Ratio measures the spatial connectedness of each predicted domain.

$\mathrm{LCCR} =\sum_{k} \frac{\left| K_{k} \right|}{n}\cdot\frac{\left| L_{k} \right|}{\left| K_{k} \right|}$.	(9)

Where $L_{k}$ denotes the largest connected component in the subgraph induced by $K_{k}$.

Moran’s I measures the spatial autocorrelation of predicted domain labels.

$$\mathrm{Moran}'s I =\sum_{k} \frac{\left| K_{k} \right|}{W}\cdot\frac{\sum_{i} \sum_{j} w_{ij}\left( x_{i}^{\left( k \right)}-\overline{x}^{\left( k \right)} \right)\left( x_{j}^{\left( k \right)}-\overline{x}^{\left( k \right)} \right)}{\sum_{i} \left( x_{i}^{\left( k \right)}-\overline{x}^{\left( k \right)} \right)^{2}}$$

(10)

Where $x_{i}^{\left( k \right)}=\mathbf{1}\left( c_{i}=K_{k} \right)$, $\overline{x}^{\left( k \right)}$ is the mean of $x^{\left( k \right)}$.

Geary’s C measures local spatial dissimilarity of predicted domain labels and is more sensitive to local label changes between neighboring spots.

$$\mathrm{Geary}'s C =\sum_{k} \frac{\left| K_{k} \right|\left( n-1 \right)}{2nW}\cdot\frac{\sum_{i} \sum_{j} w_{ij}\left( x_{i}^{\left( k \right)}-x_{j}^{\left( k \right)} \right)^{2}}{\sum_{i} \left( x_{i}^{\left( k \right)}-\overline{x}^{\left( k \right)} \right)^{2}}$$

(11)

Collectively, these metrics provide an annotation-independent assessment of whether the inferred domains are spatially coherent and well organized. Overall, higher values of Neighborhood Label Agreement, Domain Compactness, Largest Connected Component Ratio, and Moran’s I, together with lower Geary’s C, indicate better spatial domain quality.

2. **Supplementary Tables**

**Table S1.** Details of real datasets used in experiments.

| Dataset | Species | Tissue/organ | Modalities | Number of spots | Modality 1 dimensions | Modality 2 dimensions |
| --- | --- | --- | --- | --- | --- | --- |
| DBiT-seq mouse embryonic brain | Mouse | E10 brain | RNA+Protein | 1,789 | 1,789×254 | 1,789×22 |
| E13 mouse embryo | Mouse | E13 embryo | RNA+ATAC | 2,187 | 2,187×4,422 | 2,187×50 |
| Stereo-CITE-seq mouse thymus | Mouse | Thymus | RNA+ADT | 4,697 | 4,697×23,622 | 4,697×33 |

**Table S2.** Quantitative comparison of SpaHDSRL with competing methods on the first simulated dataset across six evaluation metrics.

| Method | ARI | NMI | FMI | Homogeneity | Completeness | V-measure |
| --- | --- | --- | --- | --- | --- | --- |
| **SpaHDSRL** | **0.835 ± 0.040** | **0.854 ± 0.025** | **0.872 ± 0.030** | **0.858 ± 0.030** | **0.850 ± 0.030** | **0.854 ± 0.025** |
| SpatialGlue | 0.809 ± 0.050 | 0.844 ± 0.030 | 0.851 ± 0.040 | 0.855 ± 0.030 | 0.833 ± 0.035 | 0.844 ± 0.030 |
| COSMOS | 0.724 ± 0.050 | 0.795 ± 0.025 | 0.785 ± 0.035 | 0.805 ± 0.025 | 0.785 ± 0.025 | 0.795 ± 0.025 |
| Multigrate | 0.133 ± 0.030 | 0.403 ± 0.030 | 0.408 ± 0.008 | 0.351 ± 0.035 | 0.476 ± 0.030 | 0.403 ± 0.030 |
| pyWNN | 0.033 ± 0.026 | 0.161 ± 0.040 | 0.448 ± 0.025 | 0.105 ± 0.030 | 0.354 ± 0.070 | 0.161 ± 0.040 |

**Table S3.** Quantitative comparison of SpaHDSRL with competing methods on the second simulated dataset across six evaluation metrics.

| Method | ARI | NMI | FMI | Homogeneity | Completeness | V-measure |
| --- | --- | --- | --- | --- | --- | --- |
| **SpaHDSRL** | **0.573 ± 0.016** | **0.701 ± 0.012** | **0.614 ± 0.020** | **0.661 ± 0.017** | **0.746 ± 0.018** | **0.701 ± 0.012** |
| SpatialGlue | 0.489 ± 0.017 | 0.618 ± 0.018 | 0.551 ± 0.017 | 0.559 ± 0.022 | 0.693 ± 0.021 | 0.618 ± 0.018 |
| COSMOS | 0.507 ± 0.044 | 0.620 ± 0.031 | 0.550 ± 0.042 | 0.592 ± 0.033 | 0.652 ± 0.043 | 0.620 ± 0.031 |
| Multigrate | 0.186 ± 0.015 | 0.298 ± 0.012 | 0.271 ± 0.014 | 0.266 ± 0.019 | 0.339 ± 0.014 | 0.298 ± 0.012 |
| pyWNN | 0.170 ± 0.035 | 0.264 ± 0.054 | 0.272 ± 0.034 | 0.228 ± 0.050 | 0.317 ± 0.067 | 0.264 ± 0.054 |

**Table S4.** Quantitative comparison of SpaHDSRL with competing methods on the E10 mouse embryonic brain dataset across five evaluation metrics.

| Method | Neighborhood Label Agreement | Domain Compactness | Largest Connected Component Ratio | Moran's I | Geary's C |
| --- | --- | --- | --- | --- | --- |
| **SpaHDSRL** | **0.922** | **0.846** | **1.000** | **0.913** | **0.087** |
| SpatialGlue | 0.643 | 0.504 | 0.617 | 0.598 | 0.402 |
| COSMOS | 0.879 | 0.752 | 0.923 | 0.865 | 0.134 |
| Multigrate | 0.402 | 0.235 | 0.297 | 0.322 | 0.678 |
| pyWNN | 0.342 | 0.209 | 0.273 | 0.248 | 0.752 |

**Table S5.** Quantitative comparison of SpaHDSRL with competing methods on the E13 mouse embryo dataset across five evaluation metrics.

| Method | Neighborhood Label Agreement | Domain Compactness | Largest Connected Component Ratio | Moran's I | Geary's C |
| --- | --- | --- | --- | --- | --- |
| SpaHDSRL | **0.810** | 0.414 | **0.700** | **0.788** | **0.212** |
| SpatialGlue | 0.764 | 0.295 | 0.532 | 0.717 | 0.282 |
| COSMOS | 0.802 | **0.447** | 0.665 | 0.777 | 0.223 |
| Multigrate | 0.531 | 0.349 | 0.364 | 0.464 | 0.536 |
| pyWNN | 0.280 | 0.073 | 0.128 | 0.163 | 0.836 |

**Table S6.** Quantitative comparison of SpaHDSRL with competing methods on the Stereo-CITE-seq mouse thymus dataset across five evaluation metrics.

| Method | Neighborhood Label Agreement | Domain Compactness | Largest Connected Component Ratio | Moran's I | Geary's C |
| --- | --- | --- | --- | --- | --- |
| **SpaHDSRL** | **0.776** | **0.188** | **0.733** | **0.740** | **0.260** |
| SpatialGlue | 0.549 | 0.100 | 0.335 | 0.481 | 0.519 |
| COSMOS | 0.620 | 0.058 | 0.463 | 0.549 | 0.451 |
| Multigrate | 0.390 | 0.029 | 0.233 | 0.290 | 0.709 |
| pyWNN | 0.373 | 0.068 | 0.259 | 0.272 | 0.728 |

**Table S7.** Ablation study of SpaHDSRL on the first simulated dataset.

| Model variant | ARI | NMI | FMI | Homogeneity | Completeness | V-measure |
| --- | --- | --- | --- | --- | --- | --- |
| **Full model** | **0.835** | **0.854** | **0.872** | **0.858** | **0.850** | **0.854** |
| w/o feature graph | 0.341 | 0.384 | 0.500 | 0.372 | 0.396 | 0.384 |
| w/o spatial graph | 0.367 | 0.506 | 0.518 | 0.492 | 0.520 | 0.506 |
| w/o DGI | 0.665 | 0.752 | 0.738 | 0.765 | 0.739 | 0.752 |
| w/o spatial regularization | 0.740 | 0.759 | 0.797 | 0.767 | 0.751 | 0.759 |

**Table S8.** Ablation study of SpaHDSRL on the second simulated dataset.

| Model variant | ARI | NMI | FMI | Homogeneity | Completeness | V-measure |
| --- | --- | --- | --- | --- | --- | --- |
| **Full model** | **0.573** | **0.701** | **0.614** | **0.661** | **0.746** | **0.701** |
| w/o feature graph | 0.510 | 0.658 | 0.574 | 0.591 | 0.741 | 0.658 |
| w/o spatial graph | 0.219 | 0.352 | 0.287 | 0.327 | 0.381 | 0.352 |
| w/o DGI | 0.542 | 0.671 | 0.600 | 0.611 | 0.744 | 0.671 |
| w/o spatial regularization | 0.308 | 0.474 | 0.381 | 0.429 | 0.529 | 0.474 |

**Table S9.** Ablation study of SpaHDSRL on the E10 mouse embryonic brain dataset.

| Model variant | Neighborhood Label Agreement | Domain Compactness | Largest Connected Component Ratio | Moran's I | Geary's C |
| --- | --- | --- | --- | --- | --- |
| **Full model** | **0.922** | **0.846** | **1.000** | **0.913** | **0.087** |
| w/o feature graph | 0.887 | 0.753 | 0.875 | 0.872 | 0.127 |
| w/o spatial graph | 0.189 | 0.048 | 0.116 | 0.070 | 0.929 |
| w/o DGI | 0.909 | 0.814 | 0.982 | 0.898 | 0.102 |
| w/o spatial regularization | 0.860 | 0.539 | 0.742 | 0.840 | 0.160 |

**Table S10.** Ablation study of SpaHDSRL on the E13 mouse embryo dataset.

| Model variant | Neighborhood Label Agreement | Domain Compactness | Largest Connected Component Ratio | Moran's I | Geary's C |
| --- | --- | --- | --- | --- | --- |
| Full model | **0.810** | 0.414 | **0.700** | **0.788** | **0.212** |
| w/o feature graph | 0.754 | **0.415** | 0.584 | 0.726 | 0.274 |
| w/o spatial graph | 0.323 | 0.159 | 0.209 | 0.240 | 0.759 |
| w/o DGI | 0.656 | 0.320 | 0.413 | 0.610 | 0.390 |
| w/o spatial regularization | 0.678 | 0.333 | 0.473 | 0.641 | 0.359 |

**Table S11.** Ablation study of SpaHDSRL on the Stereo-CITE-seq mouse thymus dataset.

| Model variant | Neighborhood Label Agreement | Domain Compactness | Largest Connected Component Ratio | Moran's I | Geary's C |
| --- | --- | --- | --- | --- | --- |
| **Full model** | **0.776** | **0.188** | **0.733** | **0.740** | **0.260** |
| w/o feature graph | 0.673 | 0.087 | 0.466 | 0.618 | 0.382 |
| w/o spatial graph | 0.480 | 0.058 | 0.377 | 0.393 | 0.607 |
| w/o DGI | 0.752 | 0.159 | 0.554 | 0.702 | 0.297 |
| w/o spatial regularization | 0.683 | 0.047 | 0.512 | 0.626 | 0.374 |

**Table S12.** Quantitative comparison of single-modality controls and integrated SpaHDSRL on real datasets.

| Dataset | Setting | Neighborhood Label Agreement | Domain Compactness | Largest Connected Component Ratio | Moran's I | Geary's C |
| --- | --- | --- | --- | --- | --- | --- |
| E10 mouse embryonic brain dataset | **Integrated** | **0.922** | **0.846** | **1.000** | **0.913** | **0.087** |
|  | RNA-only | 0.870 | 0.714 | 0.836 | 0.855 | 0.145 |
|  | Protein-only | 0.376 | 0.213 | 0.177 | 0.296 | 0.703 |
| E13 mouse embryo dataset | **Integrated** | **0.810** | **0.414** | **0.700** | **0.788** | **0.212** |
|  | RNA-only | 0.726 | 0.388 | 0.544 | 0.693 | 0.308 |
|  | ATAC-only | 0.646 | 0.284 | 0.390 | 0.599 | 0.401 |
| Stereo-CITE-seq mouse thymus dataset | **Integrated** | **0.776** | **0.188** | **0.733** | **0.740** | **0.260** |
|  | RNA-only | 0.622 | 0.083 | 0.406 | 0.553 | 0.447 |
|  | ADT-only | 0.699 | 0.108 | 0.527 | 0.651 | 0.349 |

**Table S13.** Sensitivity analysis of KNN neighborhood size in cross-modal neighbor aggregation on the first simulated dataset.

| k | Mean similarity | Median similarity | Minimum similarity | 5th percentile similarity | ARI | NMI | FMI | Homogeneity | Completeness | V-measure |
| --- | --- | --- | --- | --- | --- | --- | --- | --- | --- | --- |
| 3 | 0.924 | 0.924 | 0.916 | 0.920 | 0.849 | 0.865 | 0.883 | 0.875 | 0.857 | 0.866 |
| 5 | 0.910 | 0.913 | 0.876 | 0.882 | 0.835 | 0.854 | 0.872 | 0.858 | 0.850 | 0.854 |
| 10 | 0.877 | 0.876 | 0.851 | 0.859 | 0.6817 | 0.766 | 0.751 | 0.777 | 0.755 | 0.766 |
| 15 | 0.819 | 0.813 | 0.779 | 0.791 | 0.646 | 0.743 | 0.723 | 0.755 | 0.732 | 0.743 |
| 30 | 0.756 | 0.756 | 0.731 | 0.738 | 0.532 | 0.655 | 0.635 | 0.662 | 0.649 | 0.655 |

**Table S14.** Sensitivity analysis of downstream KNN graph size in clustering performance on the first simulated dataset.

| k | ARI | NMI | FMI | Homogeneity | Completeness | V-measure |
| --- | --- | --- | --- | --- | --- | --- |
| 10 | 0.851 | 0.862 | 0.885 | 0.860 | 0.863 | 0.862 |
| 25 | 0.849 | 0.859 | 0.884 | 0.857 | 0.861 | 0.859 |
| 50 | 0.835 | 0.854 | 0.872 | 0.858 | 0.850 | 0.854 |
| 75 | 0.908 | 0.900 | 0.929 | 0.904 | 0.896 | 0.900 |
| 90 | 0.902 | 0.896 | 0.924 | 0.901 | 0.891 | 0.896 |

**Table S15.** Sensitivity analysis of the spatial regularization strength $\lambda$ on the first simulated dataset.

| $\lambda$ | ARI | NMI | FMI | Homogeneity | Completeness | V-measure |
| --- | --- | --- | --- | --- | --- | --- |
| 0 | 0.740 | 0.759 | 0.797 | 0.767 | 0.751 | 0.759 |
| 0.01 | 0.687 | 0.758 | 0.773 | 0.722 | 0.796 | 0.758 |
| 0.05 | 0.835 | 0.854 | 0.872 | 0.858 | 0.850 | 0.854 |
| 0.08 | 0.862 | 0.870 | 0.893 | 0.878 | 0.862 | 0.870 |
| 0.10 | 0.686 | 0.787 | 0.755 | 0.798 | 0.776 | 0.787 |

**Table S16.** Sensitivity analysis of the clustering number on the E10 mouse embryonic brain dataset.

| Clustering number | Neighborhood Label Agreement | Domain Compactness | Largest Connected Component Ratio | Moran's I | Geary's C |
| --- | --- | --- | --- | --- | --- |
| 6 | 0.949 | 0.720 | 1.000 | 0.937 | 0.064 |
| 8 | 0.931 | 0.822 | 1.000 | 0.920 | 0.080 |
| 10 | 0.922 | 0.846 | 1.000 | 0.913 | 0.087 |
| 12 | 0.916 | 0.878 | 1.000 | 0.908 | 0.092 |

**Table S17.** Effect of modality-order swapping on the first simulated dataset.

| Modality order | ARI | NMI | FMI | Homogeneity | Completeness | V-measure |
| --- | --- | --- | --- | --- | --- | --- |
| Original order | 0.835 | 0.854 | 0.872 | 0.858 | 0.850 | 0.854 |
| Swapped order | 0.868 | 0.877 | 0.897 | 0.885 | 0.868 | 0.877 |

**Table S18.** Effect of modality-order swapping on the E10 mouse embryonic brain dataset.

| Model variant | Neighborhood Label Agreement | Domain Compactness | Largest Connected Component Ratio | Moran's I | Geary's C |
| --- | --- | --- | --- | --- | --- |
| Original order | 0.922 | 0.846 | 1.000 | 0.913 | 0.087 |
| Swapped order | 0.883 | 0.683 | 0.961 | 0.869 | 0.131 |

3. **Supplementary Figures**

**Figure S1.** Sensitivity analyses of key SpaHDSRL parameters and visualization of learned gating weights on the first simulated dataset. (A) Spatial domain results obtained under different aggregation neighborhood sizes k. (B, C) Similarity distribution of selected top-k neighbors under different aggregation k values. (D) Clustering performance under different aggregation k values. (E) Spatial domain results obtained using different KNN graph sizes in the downstream Leiden clustering. (F) Clustering performance under different downstream Leiden KNN settings. (G) Spatial domain results under different spatial regularization strength $\lambda$ values. (H) Clustering performance under different spatial regularization strength $\lambda$ values. (I) Spatial distributions of learned intra-modality spatial-graph weights for each modality. (J) Spatial distributions of learned cross-modality gating weights for each modality.

**Figure S2.** Modality-order analysis and ablation studies of SpaHDSRL on simulated datasets. (A) Ground-truth spatial domains of the first simulated dataset. (B) Spatial clustering and UMAP visualization under the modality-swapped setting, where ADT was used as the first modality. (C) Spatial domain results of the full SpaHDSRL model and ablation variants on the first simulated dataset. (D) Quantitative comparison of the full model and ablation variants on the first simulated dataset. (E) Ground-truth spatial domains of the second simulated dataset. (F) Spatial domain results of the full model and ablation variants on the second simulated dataset. (G) Quantitative comparison of the full model and ablation variants on the second simulated dataset.

**Figure S3.** Modality-order analysis, single-modality controls, ablation studies, downstream analyses, and sensitivity analysis of SpaHDSRL on the E10 mouse embryonic brain dataset. (A) Spatial clustering and UMAP visualization under the modality-swapped setting, where protein was used as the first modality. (B, C) Spatial clustering and UMAP visualizations of the RNA-only and protein-only control models. (D) Spatial domain results of the full SpaHDSRL model and ablation variants. (E) Quantitative comparison of the full model and ablation variants across five spatial evaluation metrics. (F) Heatmap of the domain-level expression patterns of all 22 proteins. (G) Dotplot of the top five differentially expressed genes for each domain. (H) Spatial domain results obtained with different target clustering numbers. (I) Clustering performance under diiferent target clustering number.

**Figure S4.** Spatial expression heatmaps of all 22 proteins in the E10 mouse embryonic brain dataset.

**Figure S5.** Single-modality controls, ablation studies, and downstream analyses of SpaHDSRL on the E13 mouse embryo dataset. (A, B) Spatial clustering and UMAP visualizations of the RNA-only and ATAC-only control models. (C) Spatial domain results of the full SpaHDSRL model and ablation variants. (D) Quantitative comparison of the full model and ablation variants across five spatial evaluation metrics (E) Spatial distributions of representative accessible tiles across the tissue. (F) Moran’s I scores of the top ten spatially autocorrelated accessible tiles. (G) Spatial visualization of Kdm1a RNA expression and ATAC-derived gene activity. (H) Dotplot of the top five differentially expressed genes for each domain.

**Figure S6.** Single-modality controls, ablation studies, and downstream analyses of SpaHDSRL on the Stereo-CITE-seq mouse thymus dataset. (A, B) Spatial clustering and UMAP visualizations of the RNA-only and ADT-only control models. (C) Spatial domain results of the full SpaHDSRL model and ablation variants. (D) Quantitative comparison of the full model and ablation variants across five spatial evaluation metrics. (E) Spatial distributions of representative ADT features across the thymus tissue. (F) Moran’s I scores of all 33 measured ADT features ranked by spatial autocorrelation (G) Heatmap of the domain-level expression patterns of all 33 measured ADT features.
